# Assessing the Adoption of Recommended Standards, Novel Approaches, and Best Practices for Animal Health Surveillance by Decision Makers in Europe

**DOI:** 10.3389/fvets.2019.00375

**Published:** 2019-11-06

**Authors:** Barbara Häsler, Maria Garza, Betty Bisdorff, Anaïs Léger, Saraya Tavornpanich, Marisa Peyre, Ann Lindberg, Gerdien van Schaik, Lis Alban, Katharina D. C. Stärk

**Affiliations:** ^1^Veterinary Epidemiology, Economics and Public Health Group, Department of Pathobiology and Population Sciences, Royal Veterinary College, London, United Kingdom; ^2^SAFOSO AG, Bern, Switzerland; ^3^Department of Aquatic Animal Health and Welfare, Norwegian Veterinary Institute, Oslo, Norway; ^4^CIRAD, UMR ASTRE, Montpellier, France; ^5^ASTRE, CIRAD, INRA, Univ Montpellier, Montpellier, France; ^6^Department of Disease Control and Epidemiology, National Veterinary Institute, Uppsala, Sweden; ^7^Epidemiology Group, Royal GD, Deventer, Netherlands; ^8^Department of Farm Animal Health, Faculty of Veterinary Medicine, Utrecht University, Utrecht, Netherlands; ^9^Risk Assessment Group, Department of Food Safety and Veterinary Issues, Danish Agriculture and Food Council, Copenhagen, Denmark; ^10^Department of Veterinary and Animal Sciences, University of Copenhagen, Frederiksberg, Denmark

**Keywords:** animal health, surveillance, standards, evaluation, disease control

## Abstract

Animal health surveillance is an important tool for disease mitigation and helps to promote animal health and welfare, protect human health, support efficient animal production, and enable trade. This study aimed to assess adoption of recommended standards and best practice for surveillance (including risk-based approaches) in Europe. It included scoping interviews with surveillance experts in Denmark, the Netherlands, Norway, and Switzerland to gather information on knowledge acquisition, decisions and implementation of surveillance, and perceptions. This was followed by an online survey among animal health and food safety surveillance users in EU, EEA, and Schengen countries. A total of 166 responses were collected from 27 countries; 111 were eligible for analysis. A strong preference for legislation and established standards was observed, with peer-reviewed publications, conferences, symposia, and workshops to be major sources of information. The majority of respondents indicated a need for international evaluation for surveillance and implied that considerations of cost-effectiveness were essential when making a decision to adopt new surveillance standards. However, most of the respondents did not use a formal evaluation to inform the adoption of new standards or only conducted a descriptive assessment before their implementation or adaptation. Only a few respondents reported a quantitative economic evaluation despite economic efficiency being considered as a highly relevant criterion for surveillance implementation. Constraints mentioned in the adoption of new surveillance standards included insufficient time, financial and human resources, and lack of competency. Researchers aiming to achieve impact by their surveillance work are advised to consider ways of influencing binding standards and to disseminate their work pro-actively using varied channels of engagement tailored to relevant target audiences and their needs. Generally, a more formal linkage between surveillance information and disease mitigation decisions—for example, by using systematic evaluation—could help increase the economic value of surveillance efforts. Finally, a collaborative, international platform for exchange and learning on surveillance as well as co-design and dissemination of surveillance standards is recommended.

## Introduction

The current European Union (EU) Animal Health Law provides enhanced opportunities to apply alternative surveillance approaches achieving comparable levels of evidence. This allows increasing economic efficiency and effectiveness of surveillance while taking into account local practices and farming conditions. It requires a shift in the design of surveillance systems toward output-based (what has to be achieved) rather than input-based approaches (which activities must be undertaken) (1). According to Article 27 of the EU Animal Health Law (2), prevention and control measures for transmissible animal diseases should be disease-specific, taking into account disease epidemiology and associated risks, as well as characteristics of the target population. The Animal Health Law also encourages the application of risk-based approaches for surveillance. Risk-based surveillance was defined by Hoinville et al. (3) as: “making use of information about the probability of occurrence and the magnitude of the biological or economical consequence of health hazards to plan, design, and/or interpret the results obtained from surveillance systems.” It seems, however, that the benefits of risk-based surveillance are not (yet) fully exploited by all beneficiaries of surveillance. A study carried out by the European Union's Seventh Framework Programme funded project Risk-Based Animal Health Surveillance Systems (RISKSUR)^1^ showed that within the 11 EU Member States and Switzerland surveyed in 2011, slightly more than half of the surveillance components used risk-based sampling (4).

Surveillance systems are usually designed following recommended standards, i.e., guidelines issued by an authority (e.g., World Organization for Animal Health, OIE; Food and Agriculture Organization of the United Nations, FAO; World Health Organization, WHO; Codex Alimentarius Commission) or by general consent ensuring that processes and/or outputs are consistent and fit for purpose. Standards issued by international bodies like the Tripartite institutions (OIE, FAO, and WHO) are often used as international references. In principle they are not legally binding, unless they have been included in a country's national legislation (5). Also, if a country is member of the World Trade Organization, these standards are referenced in the agreement on sanitary and phytosanitary measures and can become relevant in a trade dispute. International guidelines or standards are being developed and regularly updated by the issuing organizations, such as the Tripartite institutions, in a transparent and responsive procedure. For the OIE, for example, all 182 OIE Member Countries are encouraged to contribute (6). It then takes two years for the adoption of new texts in the OIE codes, during which time the texts are submitted and then circulated to the Member Countries several times. However, in case of emergencies, standards may be developed in a shorter period and once adopted by the Assembly, they are then being circulated to the Member Countries. Some standards recommended by international authorities and/or elaborated by the EU Commission are translated into directly binding legislation relevant to a country.

Communication and knowledge transfer between the different actors involved (such as academia, livestock industry, and policy makers) in the design and implementation of surveillance systems are key and thus need to be enhanced by ensuring that the latest up-to-date standards are being applied (7). In an optimal situation, a standard should be cost-effective, feasible to implement, and robust (8), as well as politically, economically, and socially acceptable to decision makers and stakeholders. In reality, this is not always achieved because of political reasoning.

Standards set by public authorities are usually referred to as technical regulations and they are mandatory in many cases. Private standards are generally voluntary (9, 10) and developed by a non-government entity (9). In practice, private standards become de facto mandatory where compliance is required for entry into certain markets. The number of private standards and their influence on trade has risen steadily, a trend which is foreseen to continue. In the field of veterinary public health, the requirements of different stakeholders in the food chain for safe food and high welfare standards have been at the forefront of this development with retailers and other private institutions increasingly determining decisions regarding public health risks and other impacts (10–12).

To set up cost-effective risk-based surveillance systems, best practices should be applied (8). These have been defined by the RISKSUR consortium as: “working practices that are good examples using state-of-the-art methods and approaches under real-life conditions.” RISKSUR developed approaches and tools for the design and evaluation of surveillance and promoted them using publicly available educational materials as well as a best practice document. The evaluation tool (13) provided guidance on how to evaluate functional, performance, and value attributes in relation to surveillance, including least-cost analysis, cost-effectiveness analysis, and cost-benefit analysis.

While it is important to use an agreed and common terminology, standards should also be flexible so that they can be adapted to accommodate local (e.g., national) needs as well as new hazards. If an international standard cannot be implemented at national level this might constitute a barrier to effective surveillance (14). Inconsistent implementation of international guidelines in countries can weaken international disease reporting and response to health risks. Moreover, comparisons of health status between countries may be hampered by large variations in implementation of surveillance and monitoring, as for example shown with regards to the monitoring of antimicrobial residues in meat in the Netherlands, Denmark, and Switzerland (15). Another barrier to implementation of standards is that data are not always available, accessible or easy to obtain. In aquaculture, for example, it is difficult to progress in the design of risk-based surveillance, due to lack, non-availability, or paucity of data (16).

Being aware of these challenges and barriers, the follow-up project to RISKSUR called risk-based surveillance for animal health in Europe (SANTERO)^2^, aimed to promote the enhancement of risk-based surveillance methods suitable for implementation across industries and countries in Europe as well as their dissemination and integration into existing surveillance routines. However, the development of surveillance methodologies is of limited value if not adopted by the target users. Hence, the aim of this study was to gather information regarding the adoption and use of recommended surveillance standards, novel approaches and best practices across EU, European Economic Area (EEA), and Schengen countries from decision-makers for surveillance and/or their technical advisors, and/or technically competent users or data analysts who design, implement, or assess surveillance. Specific objectives were to identify drivers and constraints to uptake of surveillance standards and to identify preconditions required to achieve changes in surveillance policy in EU, EEA, and Schengen countries.

## Methods

A two-step approach was used: key informant interviews (Step 1) were conducted in a scoping study to inform the design of a questionnaire-based, online survey (Step 2). Standards were defined as “something considered by an authority or by general consent as an approved model or quality that can serve as a basis of comparison across countries” (17). It could include OIE standards (e.g., OIE surveillance guide), private standards and industry guidelines (e.g., private surveillance and monitoring in the pig industry to conform with an international private standard), best practice recommendations (e.g., RISKSUR document), EU regulation (e.g., disease notification rules), and national regulation (e.g., enhanced passive surveillance rules for the UK).

### Key Informant Interviews

SANTERO collaborators conducted 12 key informant interviews in their countries, namely Switzerland (*n* = 3), Denmark (*n* = 5), the Netherlands (*n* = 2), and Norway (*n* = 2) with target interviewees being aquatic, livestock, and food safety decision-makers for surveillance and/or their technical advisors and/or technically competent users who design, implement, or evaluate surveillance. The aim of these key informant interviews was to gain an overview on the surveillance information context in those countries, the use of existing standards/guidelines, what influences the participants' actions and decisions and potential reasons for use or non-use. The interview guide included questions on three thematic areas: (1) how and where people acquire surveillance information and knowledge; (2) factors that influence the decisions to adopt surveillance standards; and (3) perceptions of implementation of standards in their institution. The full question guide can be found in Supplementary File 1. Each interviewer conducted the interview in the language of their choice and provided the answers to each question in the form of written notes in English for each interview conducted. Once all key informant interviews were complete, an interpretive summary was generated, i.e., an active interpretation of the answers given with the aim to inform the development of the survey.

### Online Survey

Next, the resulting information was used to design an online survey in English (Supplementary File 2) that was directed at decision makers for surveillance and/or their technical advisors and/or technically competent users or data analysts who design, implement, or assess surveillance across EU, EEA, or Schengen countries. The survey included questions on respondents' characteristics (in particular their role in surveillance), the use and relevance of existing standards for animal health surveillance, procedures for data, information sharing and learning (both formal and informal), drivers and hindering factors for the adoption of new surveillance standards and evaluation of surveillance. The survey was pilot tested among collaborators in the consortium and then circulated widely online and by email using the SANTERO website, the RISKSUR newsletter and professional networks of all collaborators. The survey was open from May to July 2017. As an incentive for participation, respondents were given the opportunity to enter their names for a draw of three Amazon gift vouchers. Survey responses were monitored by the authors and where low participation was observed, the collaborators engaged their professional networks by direct contact thereby encouraging survey uptake and participation in a targeted manner. Responses were considered if a respondent answered about half of the questionnaire, i.e., all questions up to (and including) the use of existing tools (apart from the tools for aquaculture which were deemed to be more specialized). Descriptive statistics were conducted using IBM SPSS statistics 24 and Microsoft Excel. Open-ended questions (e.g., further explanations given or suggestions made) were coded manually by theme or topic and summarized in an interpretative way. Direct quotes were included where deemed appropriate from respondents that had given permission to use quotes.

### Ethics

Ethical approval for the scoping interviews and the survey was requested and granted from the Royal Veterinary College, United Kingdom; approval number “URN SR2017-1049.”

## Results

### Scoping Interviews With Surveillance Stakeholders

Twelve interviews were conducted in the Netherlands, Denmark, Switzerland, and Norway with government representatives for food safety (3), government and private sector representatives for animal health including fish (4), consultants to government in animal health or food safety (2), veterinary advisors to government (3). They were all involved in the planning, design, implementation, and/or evaluation of terrestrial, aquatic or food safety surveillance in their countries.

#### Information and Knowledge Acquisition

Interviewees reported keeping up to date on surveillance standards and acquiring information about surveillance developments in several ways. All respondents reported using sources of literature, either peer-reviewed or official material about standards and guidelines produced by OIE, EU, EFSA, or national bodies to different extents. Requesting information from national disease experts was also described. The RISKSUR best practice guide (8) was known by three interviewees; two of them knew it because they had been involved in RISKSUR and one discovered it through course attendance. All respondents explained that they gained knowledge in their professional networks through: colleagues in formal international and national meetings or working groups; informal exchange during interactions with people at work, in projects, or at conferences; (formal) professional advice from experts on disease, in academia and industry; or as attendants at courses, workshops, or conferences.

Respondents in Denmark appeared to rely mainly on their institutions for data, information, and guidance, and explained that for current surveillance of food safety, their system and data produced (based on a combination of surveillance and disease experts) allowed them to be ahead of the classic sources of surveillance information such as EU and OIE guidance and Codex Alimentarius.

Opinions about the amount and ideal data differed among respondents. Some respondents were content with the quality, suitability and amount of data, while one person observed that “*more data is always desirable*.” One informant criticized the information flows and availability, observing that final outputs and results are often not circulated despite being involved in projects and discussions. One interviewee observed that the most informative activity was the development of a new approach, while another one explained that surveillance tools are often not user-friendly and cannot be applied in official places with strict firewall protection. One interviewee emphasized that a platform for information exchange (and learning purposes) should be created, because only successful stories are widely spread, while failures, problems, or negative experiences are only known by small networks.

#### Decisions and Implementation

The use of surveillance standards and best practice guidance varied widely across countries and species. Interviewees from Denmark explained that surveillance was so entrenched in the system that the use of standards was not something noticeable or to highlight. Aquatic experts declared using various standards, in particular EU legislation that establishes requirements for surveillance and diagnostics (Council Directive 2006/88), OIE guidelines for diseases not described in EU legislation or for trade with non-EU countries, and national programme standards for notifiable diseases. For surveillance to document freedom from disease in aquatic animals, an informant from Norway had consulted peer-reviewed literature to design a programme with implementation of actions based on Council Directive 2006/88.

In terrestrial animal health, export requirements by third countries were an important driver to go beyond EU legislation and OIE requirements. Technical practicality such as data availability, added value and financial means were further criteria—sometimes with the power to overrule best practice—that influenced the inclusion of new surveillance components in the system. Legislation and cost-effectiveness were regarded as the most common drivers when setting new standards or implementing new surveillance. Reluctance to change appeared to be a strong hindering factor for the adoption of new approaches or implementation of current programmes in two countries (Denmark and the Netherlands), whereas fear of remaining in a programme forever and the associated unpopularity was a strong consideration in Switzerland. Other hindering factors to implementation of best practice and standards were financial and human resources, requirements from third countries, markets, lack of knowledge and uncertainty about the effect of the change.

Decision-making processes for surveillance differed among respondents, but there was a common theme, namely that decisions are often taken in groups, and are of multidisciplinary character bringing together public, private and academic stakeholders. Differences were found in the level of formality spanning the whole spectrum from informal to mixed to formal processes and decision making.

#### Perceptions

Almost all informants stated to be satisfied with the work of their institution in the use of surveillance standards. However, several criticisms were made, and suggestions put forward for improvement. They included the need for the development and implementation of standardized approaches to promote harmonization across countries and avoid making decisions based solely on factors like political pressure, gut feeling and media influence; implementation of risk-based approaches and improvement of EU regulations in support of the approach; wider uptake of evaluation of surveillance to demonstrate effectiveness, efficiency and best practice across countries; re-consideration of passive surveillance as a valuable approach; exploring and/or enhancing models for private-public-academic partnerships; more efficient use of and access to data (in real-time if feasible); and enhancement of community-based surveillance and engagement for more pro-active information sharing. It was pointed out by several interviewees that standards for the evaluation of surveillance were missing and would be necessary.

Informants attributed differing importance to existing standards with some rating design, implementation, and evaluation as critical, whereas others attributed most importance to prioritization.

Finally, most interviewees perceived a high-quality level of outputs produced by the international surveillance community but listed critical needs. These included a need for an umbrella organization to support the international surveillance community; formal evaluation standards; surveillance standardization across countries; involvement of industry partners; and management of influencing factors (e.g., political, consumer concerns, perceptions, and emotions). Moreover, informants perceived a lack of international agreements on what high quality, fit-for-purpose surveillance constitutes. They acknowledged the existence of a wide range of contexts with differing infrastructure, capacity, political factors, consumer demands, among others, and explained that there would be many different opinions on what good quality would constitute. It was suggested that context-specific barriers to implementation of new standards should be investigated at the country level to pave the way for effective implementation.

### Survey

#### Response Rate and Respondent Characteristics

A total of 166 people started the survey, of which four did not give consent, 30 gave consent without providing any answers, 36 completed the survey partially and 96 completed the survey in full. After consideration of the inclusion threshold, 111 responses were analyzed.

**Figure 1:**
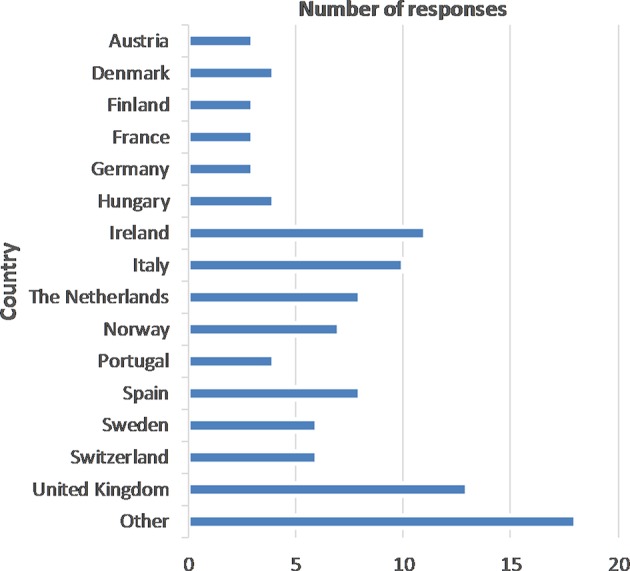
Number of survey responses by country.

The number of responses by country are presented in Figure 1. The “other” in Figure 1 includes one or two responses each received from Belgium, Bulgaria, Croatia, Czech Republic, Greece, Lithuania, Luxembourg, Malta, Poland, Romania, Slovakia. A total of 58% of respondents were from the public sector, 17% from academia, 11% from research institutes, 6% from the private sector, 4% from non-government organizations, 3% from other organizations, and 2% from small or medium sized enterprises. A total of 28% of respondents were senior researchers, 23% each from middle and upper management, 9% trained professionals, 5% each administrative staff and junior researchers, 3% junior management, and the remaining respondents (<2% each) were self-employed/partner, student, temporary employee, policy advisor, and technical officer. Surveillance responsibilities of respondents included (with a few exceptions) multiple activities with analysis of surveillance data mentioned 80 times out of 483, development of surveillance design 33/483, communication of surveillance information to decision-makers 68/483, implementation of surveillance 53/483, assessment of surveillance system performance 48/483, development of new methods for surveillance designs 45/483, decisions on whether to run a surveillance component or programme and development of new methods for surveillance evaluation 33/483 each, assessment of surveillance system value/economic efficiency 32/483, decision on resource allocation for surveillance 17/483, and other (e.g., policy advisor, methods for fraud detection, diagnostics) 9/483. With regards to species focus, 42% of respondents stated that their roles were general and not species focused. For the other respondents, multiple species were often covered. Most frequently listed were terrestrial livestock (51/134 responses), wildlife (22/134), and fish and molluscs (14/134). Bees, camelids and deer, companion animals, equidae, insect vectors, and other (e.g., humans, food safety) were listed between 6 and 11 times.

With regards to the purpose of surveillance people were in charge of, multiple answers were given by respondents. The most frequently mentioned purposes were to demonstrate absence of disease or infection (73/445), confirm disease status (69/445), identify changes in disease status to facilitate early response (67/445), provide information for assessing and managing risks (64/445), and to assess if intervention measures are efficient (monitor progress, verify success) (46/445). Other surveillance purposes were mentioned 30 times or less. When asked what hazards people were responsible for, 32% of respondents said that their role was general and did not have a specific hazard focus. Among the other respondents, multiple hazards were commonly cited with the most frequent being emerging/re-emerging infectious diseases (54/258), zoonoses (52/258), endemic infectious disease (48/258), exotic infectious disease (40/258), and antimicrobial resistance (28/258). Chemical hazards, antimicrobial use, physical hazards and other (e.g., nanoparticles) were mentioned less frequently.

#### Use of Existing Surveillance Standards

**Figure 2:**
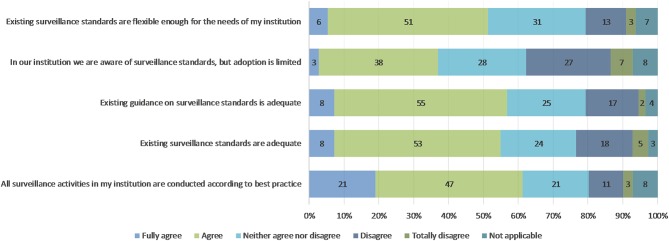
Respondents' answers to statements on quality and adoption of surveillance standards, *n* = 111.

When asked about the quality and adoption of surveillance standards (Figure 2), the majority of respondents predominantly agreed or fully agreed with the statements provided apart from the statement “in our institutions we are aware of surveillance standards, but adoption is limited,” where 41 people fully agreed or agreed and 34 totally disagreed or disagreed. A total of 52% of respondents affirmed that national government or industry standards went beyond regional (e.g., EU) or international (e.g., OIE) standards; 29% stated that they did not think so and 19% did not know.

Existing standards used by the large majority of respondents were EU legislation (89%), peer-reviewed publications (87%), national legislation (84%), and the OIE codes for terrestrial and/or aquatic species (75%) (Table 1). The other standards were used by less than half of the respondents with standards for aquatic species being used by the smallest number of people. The most frequently mentioned purposes for using most standards were planning of surveillance activities and surveillance design. For peer-reviewed literature, the most frequently mentioned purposes were surveillance data analysis and surveillance data interpretation. The OIE codes for terrestrial and aquatic species were also used frequently for diagnostic procedures for surveillance. Private standards and the FAO technical paper “surveillance and zoning for aquatic animal diseases” were frequently mentioned for the purpose of surveillance implementation. Standards were used in the majority of responses “several times a year” (median 52.3%, min 33.3%, max 85.7%). Other standards and resources that were used by respondents were ISO standards; OIE training manuals on surveillance and international reporting of diseases in wild animals; statistical, surveillance and veterinary epidemiology textbooks; “own” standards, i.e., standards adapted for application to One Health for use by the institution; standards from breeders' associations; FSA, WHO, USDA, AECOSAN, and MAPAMA standards; surveillance standards and gray literature from other countries including non-EU countries (e.g., strategic reviews or programme reports of surveillance systems in specific countries).

**Table 1:** Surveillance standards used by respondents and surveillance purpose the standards were used for.

	**Standards used**			**Surveillance purpose standard was used for (multiple answers possible)**											
	**Yes**	**No**	**No answer**	**Prioritization of hazards for surv**.	**Planning of surv. activities**	**Surv. design**	**Surv. implemen-tation**	**Diagnostic procedures for surv**.	**Surv. data analysis**	**Surv. data interpretation**	**Communication /reporting of surveillance findings**	**Evaluation of surveillance**	**Requirements for certification or accreditation**	**Other**	**Total**
EU legislation	99 (89%)	12 (11%)	0	34 (8.8%)	59 (15.2%)	54 (14%)	39 (10.1%)	41 (10.6%)	23 (5.9%)	27 (7%)	41 (10.6%)	28 (7.2%)	35 (9%)	6 (1.6%)	387
Peer-reviewed publications	97 (87%)	14 (13%)	0	33 (7.6%)	53 (12.2%)	64 (14.7%)	39 (8.9%)	49 (11.2%)	59 (13.5%)	62 (14.2%)	33 (7.6%)	40 (9.2%)	4 (0.9%)	0 (0%)	436
National legislation	93 (84%)	18 (16%)	0	26 (7.1%)	50 (13.7 %)	46 (12.6%)	40 (11%)	37 (10.1%)	28 (7.7%)	33 (9%)	43 (11.8%)	27 (7.4%)	29 (7.9%)	6 (1.6%)	365
OIE codes for terrestrial and/or aquatic species	83 (75%)	28 (25%)	0	19 (6.6%)	34 (11.8%)	48 (16.6%)	21 (7.3%)	46 (15.9%)	21 (7.3%)	23 (8%)	26 (9%)	24 (8.3%)	25 (8.7%)	2 (0.7%)	289
OIE Guide to Terrestrial Animal Health Surveillance	47 (42%)	64 (58%)	0	12 (8%)	18 (12%)	23 (15.3%)	14 (9.3%)	17 (11.3%)	15 (10%)	12 (8%)	15 (10%)	11 (7.3%)	12 (8%)	1 (0.7%)	150
Private standards	31 (28%)	80 (72%)	0	10 (8.8%)	15 (13.2%)	13 (11.4%)	14 (12.3%)	11 (9.6%)	8 (7%)	14 (12.3%)	11 (9.6%)	7 (6.1%)	8 (7%)	3 (2.6%)	114
Codex Alimentarius	31 (28%)	80 (72%)	0	12 (12%)	15 (15%)	9 (9%)	6 (6%)	7 (7%)	10 (10%)	13 (13%)	10 (10%)	9 (9%)	6 (6%)	3 (3%)	100
FAO risk-based disease surveillance manual	30 (27%)	81 (73%)	0	10 (9.5%)	17 (16.2%)	24 (22.9%)	9 (8.6%)	7 (6.7%)	9 (8.6%)	7 (6.7%)	10 (9.5%)	8 (7.6%)	4 (3.8%)	0 (0%)	105
RISKSUR best practice document	21 (19%)	90 (81%)	0	7 (8.4%)	14 (16.9%)	17 (20.5%)	8 (9.6%)	4 (4.8%)	10 (12%)	7 (8.4%)	5 (6%)	11 (13.3%)	0 (0%)	0 (0%)	83
Book “Epidemiological surveillance in animal health”	14 (13%)	97 (87%)	0	4 (9.1%)	5 (11.4%)	10 (22.7%)	4 (9.1%)	4 (9.1%)	5 (11.4%)	4 (9.1%)	3 (6.8%)	4 (9.1%)	0 (0%)	1 (2.3%)	44
OIE Guide for Aquatic Animal Health surveillance	7 (6%)	90 (81%)	14 (12%)	2 (6.1%)	6 (18.2%)	6 (18.2%)	3 (9.1%)	3 (9.1%)	2 (6.1%)	1 (3%)	3 (9.1%)	4 (12.1%)	3 (9.1%)	0 (0%)	33
Survey toolbox for aquatic animal diseases: a practical manual and software package	5 (5%)	92 (83%)	14 (12%)	0 (0%)	1 (5.6%)	5 (27.8%)	2 (11.1%)	2 (11.1%)	3 (16.7%)	3 (16.7%)	1 (5.6%)	1 (5.6%)	0 (0%)	0 (0%)	18
FAO technical paper “surveillance and zoning for aquatic animal diseases”	4 (4%)	93 (84%)	14 (12%)	2 (13.3%)	3 (20%)	2 (13.3%)	3 (20%)	2 (13.3%)	2 (13.3%)	1 (6.7%)	0 (0%)	0 (0%)	0 (0%)	0 (0%)	15

*Surv, surveillance*.

**Figure 3:**
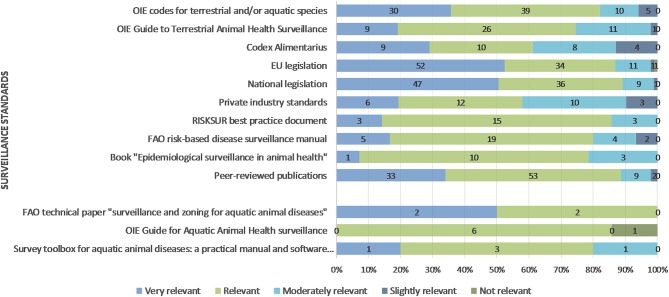
Relevance of standards for the respondents' surveillance work.

The relevance of the standards for respondents' surveillance work was mostly deemed very relevant, relevant or moderately relevant; only a minority of respondents stated a slight or no relevance (Figure 3).

#### Information and Data Exchange

When enquiring about procedures for data and information sharing as well as learning (formal and informal) (Table 2), the most regularly used international sources were the exchange with international colleagues, international scientific publications, and international conferences or symposia. Sources used less often were international online courses and official communications by private standard setting bodies. For the national sources, most frequently used were exchange with colleagues at the workplace, exchange with national colleagues outside the workplace, and collaboration in national surveillance research or projects. The least frequently used national source were national online courses.

**Table 2:** Respondents' frequency of using national and international sources to learn about new surveillance standards or best practice for surveillance; *n* = 107.

	**Very often**	**Often**	**Sometimes**	**Rarely**	**Never**
**NATIONAL**					
National conference or symposium	9 (8.4%)	31 (29%)	43 (40.2%)	15 (14%)	3 (2.8%)
Scientific national publications	9 (8.4%)	30 (28%)	37 (34.6%)	22 (20.6%)	4 (3.7%)
Lay national publications	9 (8.4%)	17 (15.9%)	27 (25.2%)	26 (24.3%)	20 (18.7%)
Official communications by private standard setting bodies	3 (2.8%)	11 (10.3%)	29 (27.1%)	30 (28%)	24 (22.4%)
Official communications by national public bodies	19 (17.8%)	34 (31.8%)	27 (25.2%)	15 (14%)	7 (6.5%)
National, non-institutional training event	0 (0%)	12 (11.2%)	29 (27.1%)	35 (32.7%)	26 (24.3%)
Institutional training event	4 (3.7%)	21 (19.6%)	39 (36.4%)	20 (18.7%)	18 (16.8%)
Exchange with colleagues at my workplace	38 (35.5%)	44 (41.1%)	16 (15%)	6 (5.6%)	1 (0.9%)
Exchange with national colleagues outside my workplace	20 (18.7%)	41 (38.3%)	30 (28%)	8 (7.5%)	3 (2.8%)
Collaboration in national surveillance research or projects	11 (10.3%)	40 (37.4%)	32 (29.9%)	14 (13.1%)	5 (4.7%)
National online courses	0 (0%)	7 (6.5%)	18 (16.8%)	28 (26.2%)	41 (38.3%)
**INTERNATIONAL**					
International conference or symposium	7 (6.5%)	29 (27.1%)	48 (44.9%)	20 (18.7%)	2 (1.9%)
International scientific publications	22 (20.6%)	44 (41.1%)	34 (31.8%)	5 (4.7%)	0 (0%)
International lay publications	3 (2.8%)	22 (20.6%)	37 (34.6%)	28 (26.2%)	10 (9.3%)
Official communications by OIE or FAO	5 (4.7%)	23 (21.5%)	41 (38.3%)	23 (21.5%)	9 (8.4%)
Official communications by private standard setting bodies	3 (2.8%)	8 (7.5%)	25 (23.4%)	39 (36.4%)	25 (23.4%)
EU bulletin	7 (6.5%)	17 (15.9%)	29 (27.1%)	30 (28%)	20 (18.7%)
International training event	2 (1.9%)	15 (14%)	40 (37.4%)	29 (27.1%)	18 (16.8%)
Exchange with international colleagues outside my workplace	7 (6.5%)	38 (35.5%)	41 (38.3%)	15 (14%)	3 (2.8%)
Collaboration in international surveillance research or projects	9 (8.4%)	20 (18.7%)	34 (31.8%)	29 (27.1%)	10 (9.3%)
International online courses	0 (0%)	6 (5.6%)	27 (25.2%)	42 (39.3%)	28 (26.2%)

When asked whether they felt sufficiently informed about existing surveillance standards and best practice, 59/107 of respondents affirmed, 30/107 said no, and 18/107 did not know. Among those who said no and gave an explanation of what was missing, reasons cited included issues related to time (“*never enough time to learn about everything that is out there”*), difficulties to have an overview of everything (“*difficult to have an overview, learnt about new ones through this questionnaire“*) and too many sources to use (“*it is difficult to get an overview because of the amount of sources”*). Also, respondents pointed out topics that did not receive enough attention including guidelines for passive surveillance, non-statutory surveillance, common coding and parametric language, and design prevalence to prove freedom of certain diseases. Several suggestions were made on how people could be better informed including the coordination of sources, i.e., creation of a network, mailing list, website, forum, or regular gathering with information about surveillance standards, face-to-face or online training, or using national reference laboratories or an international bulletin for dissemination of information.

When asked whether they received information on new surveillance standards and best practice in a timely manner, 49/107 respondents said yes, 35/107 said no, and 23/107 did not know. Barriers mentioned included a lack of coordination; absence of a central (single) platform, group, association or mechanism with a (formal or coordinated) procedure for critical review and regular dissemination (e.g., with newsletters or other forms of notification), and a lack of open source data or information. Several respondents described difficulties related to having to search actively for information, the number of sources to consult, the time required to do so, and the challenge to decide what information to consider. For example: “*There are too many different web pages for standards and lot of standards. It is time consuming to study every single document and to decide to follow it or not.”* A suggestion made by several respondents to tackle these challenges included the creation of a central repository or platform (termed by one respondent as “*knowledge bank*”) including information on the quality and applicability of the different standards and an information dissemination mechanism with the possibility to subscribe for regular updates (e.g., email list, social media groupings). Other suggestions included the provision of practical training sessions, more regular exchange between different stakeholders, generation of applied examples/case studies, and elaboration of coding standards for surveillance. A few respondents stated that existing dissemination channels (e.g., EUR-Lex) were effective and that they did not have a need for improvement.

#### Evaluation of Surveillance, Drivers, and Hindering Factors

A total of 75/99 of respondents said yes to the question whether there was a need for international evaluation standards for surveillance; 7/99 said no and 17/99 did not know. When asked to explain why they had given this answer, the most frequent answer (given by 41 people) related to the need for standardization, harmonization, and comparison of evaluation outcomes across countries (e.g., “*To enable comparison and help understand surveillance results from other countries”* or “*To be able to compare surveillance performance and efficiency across countries”*). Five people stated that the evaluation standards in place and evaluations conducted were already good enough (e.g., “*there is already enough guidance for evaluation of surveillance”*). Six people described the importance for risk mitigation and achievement of health outcomes such as animal and public health and food safety (e.g., “*These standards are necessary to control animal health and food safety world wide”*). Four people pointed out the importance of learning from other countries' or people's experiences (e.g., “*I think it would be helpful as a way to learn from others; if we have the same task, how do we solve it in our own context?”*). Evidence for trade partners and the ability to enable trade was mentioned three times. Other (single) responses included benchmarking, evaluation of national standards, and improvement of capacity and expertize. One person stated that standards were too general for varied contexts: “*Most of the time standards are to (sic) general to be useful in specific situations. What is needed is deep teoretical (sic) knowledge and experience to approaches.”*

When asked about who should be in charge of developing evaluation standards for surveillance, OIE (64/237), the scientific community (55/237), and the EU (53/237) were selected most often, followed by the FAO (24/237). Several respondents also suggested other possibilities including a combination of multiple institutions and people (e.g., “*all the main stakeholders”*), national authorities, and the WHO. For the question “In your opinion, what are the three principal subject matters that such surveillance evaluation standards should cover?,” a wide range of answers was provided. The single most frequently listed item related to economic efficiency (e.g., cost-effectiveness, cost-efficiency, economics, costs, cost-benefits, economic implications). Also frequently mentioned were the types of hazards and topics to focus on (e.g., zoonoses, antimicrobial resistance, food safety, food fraud, epizootic disease, endemic disease) and surveillance attributes. Among the latter, most often mentioned were effectiveness, sensitivity, timeliness, and representativeness. Several persons suggested standards on methods for different surveillance activities (e.g., sampling or testing procedures) and design of surveillance, as well as an agreed conceptualization of the surveillance aim and purpose such as possibility for action, early warning and prevention. Answers focusing more specifically on the evaluation process suggested having guidance and/or an evaluation framework to help focus and design an evaluation (4/80 respondents) including guidance on suitable approaches (1/80), agreed metrics (1/80), minimum requirements (1/80), interpretation of evaluation results (2/80), documentation and reporting (2/80) including evaluation visualization (1/80), and communication (3/80). One person suggested the use of coding language to capture attributes and tiered data engines for analysis.

Asked about how relevant considerations of cost-effectiveness were when making a decision to adopt new surveillance standards, 79/99 deemed them to be absolutely essential or very important, whereas 18/99 said that they were of average importance and 2/99 of little importance. A total of 39/99 respondents stated that they did not conduct a formal evaluation to assess the economic efficiency when a new surveillance standard becomes available. Among those who conducted a formal evaluation, quantitative assessment of costs of the change in surveillance, quantitative assessment of the effectiveness of the change in surveillance (e.g., timeliness, sensitivity, acceptability), quantitative cost-benefit analysis, descriptive assessment of consequences, and descriptive assessment of costs were all used with similar frequency (between 21/141 and 31/141). Only quantitative cost-effectiveness analysis was used less (8/141).

**Figure 4:**
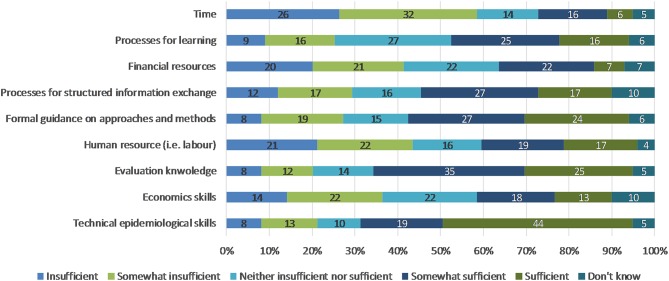
Respondents' rating of the availability of resources for the adoption of new surveillance standards in their institution.

When asked about the availability of resources for the adoption of new surveillance standards in their institutions, respondents indicated that there were largely sufficient or somewhat sufficient resources in terms of learning processes, information exchange, formal guidance on approaoches and methods, epidemiological skills and evaluation knowledge (Figure 4). Resources that were largely considered to be insufficient or somewhat insufficient were time, financial, human resource (i.e., labor) and economics skills. The question about rating the availability of resources for the adoption of evaluation standards for surveillance yielded very similar results.

## Discussion

The value of enhanced surveillance approaches and setting up risk-based surveillance systems can be realized when adopting best new practices and standards. This study showed that there is a substantial heterogeneity in the use and adoption of recommended surveillance standards, novel approaches and best practices among users from EU, EEA, and Schengen countries. Further, advancing on the findings from the RISKSUR project, it provides insights on the acquisition and use of information and available tools for decision-making, on drivers and barriers for their implementation and considerations for the development of new standards (in particular for evaluation of surveillance).

This study provided a clear picture of the standards most commonly used by respondents with EU and national legislations, peer reviewed publications, and OIE standards found to be core sources of information for most users. The dominant use of EU legislation was to be expected given that the target respondents were from EU, EEA, and Schengen countries. Other available tools including the RISKSUR best practice document were used only by a minority of respondents. An explanation of this may be that the reported information overload among users might be driving the high reported use of certain standards. Information overload can occur when the requirements needed to process (information needed to complete a task) are larger than the capacity available to process the information (18–20) which may lead to arbitrary information analysis (e.g., omission of information and being highly selective) and sub-optimal decision making (20). One possible strategy of being highly selective may be to rely more heavily on standards that are legally binding or of international importance. A wide range of countermeasures for information overload have been described in the literature; e.g., intelligent information management, decision support system, measurement system of information quality, intelligent interfaces, and coordination through inter-linked units (20). The implementation of such measures could be addressed in a coordinated, central platform—as requested by many respondents—with a mechanism for quality control and timely dissemination of information. Such a mechanism would also allow reaching target groups more effectively and thereby stimulate the uptake of new knowledge and innovation. For such a platform to be effective, clear leadership, maintenance as well as continual monitoring and recommendation are needed (21). The development of the platform would start ideally with a wide-reaching consultation to describe in depth the needs and preferences of potential users (21, 22) who may be different in the way they assimilate information or their professional needs (e.g., technical vs. decision-making requirements); this would allow to design a platform that is user-centered (21, 23). Moreover, to increase utility, it should also be open access with easily accessible and interpretable content (23). Because the development, adoption and use of standards is a cyclical process, the platform should also have a function to assimilate information from its users to produce a system where information flows back and forth (21)—essentially a process of co-design and co-production of knowledge with collaboration between stakeholders.

While the external validity of the findings results is limited by the geographical boundaries of the study, some findings may also apply to non-European countries. The findings in this study are generally in line with the outcome of a survey conducted by the OIE prior to its 86th General Session in 2018, as part of a technical item addressing the implementation of its standards. The study, directed at OIE members, showed a high level of support for implementing OIE standards, but identified key challenges including a lack of technical expertise among Member States and pointed toward the need of training to further facilitate their uptake (24). One of the more overarching outcomes of that discussion was the launch of the OIE Observatory project, an implementation monitoring function that will assist the OIE in ensuring that its standards are relevant and fit for purpose, and to develop a more strategic focus to its capacity building activities (25). This is also in line with other developments: The challenge of the “know-do-gap,” or bridging the gap between research and implementation, is gaining increased recognition; the WHO has even identified it as one of the most influential contemporary challenges (26). Consequently knowledge utilization or implementation science is gaining momentum to avoid the costs associated with underutilized knowledge. Multiple activities are suggested by the WHO to promote knowledge translation including various ways to exchange, share, and promote knowledge supported by dynamic learning networks (26).

To avoid duplication of efforts and increase efficiency by using data (and communication channels) that already exist (27), a dissemination, development, learning, and exchange platform as suggested above could be generated in a joint effort by international organizations (e.g., the Tripartite), legislators (e.g., EU commission), implementers (e.g., national government institutions), and researchers. Bringing partners together in this way would promote cumulative knowledge and enhance capacity building (27). Regular coordination meetings, active dissemination of new information including training could be linked to relevant international conferences such as the International Conference on Animal Health Surveillance (ICAHS), the International Symposium of Veterinary Epidemiology and Economics (ISVEE), or the International Society for Economics and Social Sciences of Animal Health (ISESSAH).

While such mechanisms to create more capacity are being designed and/or implemented, people developing new approaches, tools or guidelines may want to reflect on best ways to disseminate knowledge effectively. For effective dissemination of research findings, it has been recommended to consider impact pathways, elaborate a clear description of the target audience, select a range of dissemination channels in line with the target audience, and consider viability and funding issues (21, 27, 28). Respondents in this study acquired information on surveillance standards occasionally to very regularly from scientific and lay publications, conferences, workshops or courses, exchange with colleagues, collaboration, and official communications, i.e., they used a multitude of different channels. Because target users have different preferences for communication channels, it is recommended that researchers elaborate dissemination plans and identify pathways to reach stakeholders (e.g., practitioners, policy makers) via the different media in the short, medium, and long term (21). Importantly, social media in particular, but also other channels, should not just be advocated because they appear to be popular, but be used in a targeted way with careful audience selection, message formatting, and delivery to achieve the desired effects (22). For example, Kapp et al. (29) showed how twitter could be effective in dissemination of information to policy makers following an analysis of target users and their twitter use.

When elaborating such dissemination plans, pathways on how to get standards into legislation or OIE standards (including evaluation standards) may be considered which would necessitate effective collaborations and networking. Also, continued research on the topic can have an impact, as implied by one respondent “*This survey, was actually good to increase the awareness of different sources to search for information. However, the links to the different sources and specific matters might be merged together in one internet page which needs to be regularly updated*.” The idea of peer-learning could also be extended to the country level by making descriptions of animal health surveillance activities and their evaluations publicly available. In order to promote a more consistent approach to communication of animal health surveillance activities and their outputs, for the benefit of stakeholders, trade partners, decision makers, and risk assessors, a set of Animal Health SUrveillance guiDelines (AHSURED) have been developed, partly within SANTERO, and partly in an EFSA funded project (HOTLINE). The full AHSURED checklist with detailed item descriptions can be accessed on https://github.com/SVA-SE/AHSURED/wiki.

The survey underlined the importance of using economic evaluation criteria when planning the adoption of new standards, but only a bit more than a third of respondents indicated to conduct a formal financial or economic evaluation. Common constraints mentioned were a lack of human, time and financial resources as well as a shortage of economics skills. Consequently, new evaluation standards for surveillance that include economic evaluation guidance would need to be accompanied by knowledge transfer and capacity building. There is a role for governments to support the implementation of this process by enabling training opportunities, promoting innovation and making resources available within their institutions for capacity building as well as evaluation of surveillance. Moreover, tertiary education systems may want to consider integrating more economics into basic animal health and surveillance training. With enhanced (economic) evaluation capacity and skills, surveillance planners, implementers, and evaluators will have new tools at their disposal to create inventive evaluation and surveillance designs with limited resources.

## Data Availability Statement

The datasets generated for this study are available on request from the corresponding author.

## Ethics Statement

The studies involving human participants were reviewed and approved by the Social Science Research Ethical Review Board, Royal Veterinary College. The patients/participants provided their written informed consent to participate in this study.

## Author Contributions

All authors contributed to the conceptualization of the study, designed the interview guide, and survey and actively disseminated the survey. LA, ST, ALé, and GS performed the interviews with decision makers. MG and BH analyzed the interviews qualitatively and results from the surveys. BH and BB wrote the first draft of the manuscript. All the authors contributed to reviewing and revising the full manuscript.

### Conflict of Interest

KS and ALé were employed by the company SAFOSO. The industry-funded work conducted by SAFOSO all has a technical focus and complies with quality standards applied in academia. GS was employed by Royal GD. All the work conducted by Royal GD complied with academic quality standards. LA was employed by the Danish Agriculture & Food Council, which is an organization that gives advice to farmers and abattoirs in the Danish livestock and meat industry. The remaining authors declare that the research was conducted in the absence of any commercial or financial relationships that could be construed as a potential conflict of interest. The reviewer JF declared a past co-authorship with one of the authors KS to the handling editor.

## Footnotes

^1^https://www.fp7-risksur.eu/

^2^https://santero.fp7-risksur.eu/
