# Supplementary material for: Assessing the Adoption of Recommended Standards, Novel Approaches, and Best Practices for Animal Health Surveillance by Decision Makers in Europe
Source: Front Vet Sci. 2019 Nov 6;6:375. doi: 10.3389/fvets.2019.00375 (PMC6851048; doi:10.3389/fvets.2019.00375)
Supplement: Supplementary file 1 [file Data_Sheet_1.PDF]

## SUPPLEMENTARY FILE 1:

## QUESTION GUIDE ON ADOPTION OF RECOMMENDED SURVEILLANCE STANDARDS

These questions serve as guidance for a discussion with surveillance decision-makers, advisors and/or users in the SANTERO countries. They do not need to be followed in exactly this order. The aim of this first round is to gain an overview of the knowledge of standards, sources of information and how people take decisions on their implementation. The questions refer to standards for surveillance prioritisation, planning, design, implementation and evaluation.

**Information / knowledge acquisition**

1. How do you/your team stay informed on state-of-the-art/best practice surveillance methods and approaches?

2. Where do you commonly get your information (sources, channels) on state-of-the-art/best practice surveillance methods and approaches?
    a. Do you find these information sources and channels sufficient and adequate and why?
        i. If no, what are the critical gaps in your view?

3. Which three information sources do you find most useful/effective and why?

4. Are you aware of the RISKSUR best practice document?
    a. If yes, have you consulted it?
    b. If yes, was there guidance that you found particularly useful for your surveillance prioritisation, planning, design, implementation and/or evaluation?
    c. If no, can you think of reasons why you did not hear about it?

**Decisions and implementation**

5. How do you/your team take decisions on which novel/improved standards to adopt (description of the process)?
    a. Is this process formal or informal?
    b. Who are the people who drive this process?
    c. Are the decisions taken at the individual, team/group or institutional level (or another level)?

6. What are the key driving factors to implement novel guidance / best practice standards?

7. What are the key hindering factors that prevent the implementation of novel guidance / best practice standards?

**Perceptions**

8. Are you satisfied with your institution’s use of surveillance standards and why?
    a. If you are dissatisfied, what activities would you like to see happening to improve the uptake of best practice standards?
    b. If you are satisfied, are there activities you would like to see happening to facilitate the further uptake of surveillance standards?

9. Are there differences when it comes to the use of standards for surveillance prioritisation, planning, design, implementation and evaluation?
    a. If yes, what are these differences?
    b. If yes, what do you think are the reasons for these differences?
    c. If no, does your institution have processes in place to ensure a balanced approach? Can you describe them?
    d.
10. Do you think the international surveillance community produces high quality standards and why?
    a. If no, what are the key gaps?
    b. If yes, are there topics that you think need more attention?
