# Supplementary material for: Assessing the Adoption of Recommended Standards, Novel Approaches, and Best Practices for Animal Health Surveillance by Decision Makers in Europe
Source: Front Vet Sci. 2019 Nov 6;6:375. doi: 10.3389/fvets.2019.00375 (PMC6851048; doi:10.3389/fvets.2019.00375)
Supplement: Supplementary file 2 [file Data_Sheet_2.PDF]

SANTERO

Survey on animal health surveillance standards by Santero (http://santero.fp7-risksur.eu/)

Introduction and consent

Dear Sir, Madam,

You are being invited to participate in an online survey for a study that aims to identify drivers and constraints to surveillance uptake and to identify pre-conditions required to achieve change in surveillance policy. This activity takes place as part of the international project "SANTERO: risk-based Surveillance for ANimal healTh in EuROpe" (http://santero.fp7-risksur.eu). Santero is an international consortium that aims to promote the enhancement of risk-based surveillance methods suitable for implementation across industries and countries in Europe as well as their dissemination and integration into existing surveillance routines.

The following questionnaire has been designed to gather information regarding the current progress of adoption of recommended surveillance standards, novel approaches and best practices across EU, EEA and Schengen countries from decision-makers for surveillance and/or their technical advisors, and/or technically competent users or data analysts who design, implement, or assess surveillance. The data obtained in this questionnaire and other identifying details will be stored in a secure database and analysed by members of the project only. The findings of the research will be written up as feedback for you, for researchers and for other organisations interested in our work and be published. The written work may include quotations from the interviews, but individuals will not be named. We are happy to give you the option to review your quotation before publication if you desire so.

By accepting to participate in this survey you consent that the information provided in this questionnaire may be used in anonymous format for internal reports within the University of London and the different partners of the Santero consortium, and in documents that will be in the public domain such as external reports and published scientific research papers.

Should you have any questions, please contact the persons in charge of this activity from the Royal Veterinary College, London, UK, namely Maria Garza (mgarza3@rvc.ac.uk) and Barbara Haesler (bhaesler@rvc.ac.uk).

Note: You can exit this survey at any point and continue later if you are using the same IP address. To do so, press the exit button or close the tab.

* Do you accept to participate in this survey?

- Yes
- No

SANTERO

Survey on animal health surveillance standards by Santero (http://santero.fp7-risksur.eu/)

Respondent information

* In which country do you work?

* What type of organisation do you work for primarily?

- Academia
- Public sector (e.g. government)
- Private sector (e.g. livestock industry)
- Non-government organisation
- Research institute
- Small/medium sized enterprise
- Other (please specify)

* Which of the following best describes your current role?

- ◯ Upper management
- ◯ Middle Management
- ◯ Junior Management
- ◯ Researcher
- ◯ Self-employed/Partner
- ◯ Administrative Staff
- ◯ Support Staff
- ◯ Trained Professional
- ◯ Temporary Employee
- ◯ Student
- ◯ Other (please specify)

* Which surveillance activities are your responsible for? Tick as many as apply

- ☐ Decisions on resource allocation for surveillance
- ☐ Decisions on whether to run a surveillance component or programme
- ☐ Development of surveillance design
- ☐ Development of new methods for surveillance designs
- ☐ Development of new methods for surveillance evaluation
- ☐ Implementation of surveillance
- ☐ Analysis of surveillance data
- ☐ Assessment of surveillance system performance
- ☐ Assessment of surveillance system value/economic efficiency
- ☐ Communication of surveillance information to decision-makers
- ☐ Other (please specify)

* What species are you in charge of? Tick as many as apply.

- ☐ My role is general, does not have a species focus
- ☐ Bees
- ☐ Camelids and Deer
- ☐ Companion animals
- ☐ Fish and molluscs
- ☐ Equidae
- ☐ Insect vectors
- ☐ Terrestrial livestock
- ☐ Wildlife
- ☐ Other (please specify)

* What is/are the predominant surveillance purpose(s) you are in charge of? Tick as many as apply.

- ☐ Facilitation of trade
- ☐ Management of disease outbreaks
- ☐ How to prioritise surveillance and control measures for different health hazards
- ☐ Whether existing control measures should be maintained, stopped, or changed to improve the efficiency of surveillance and risk mitigation
- ☐ Other (please specify)

* What type(s) of hazards are you responsible for? Tick as many as apply.

- [ ] My role is general and does not have a hazard focus
- [ ] Emerging / re-emerging infectious disease
- [ ] Exotic infectious disease
- [ ] Endemic infectious disease
- [ ] Zoonoses
- [ ] Chemical hazards
- [ ] Physical hazards
- [ ] Antimicrobial use
- [ ] Antimicrobial resistance
- [ ] Other (please specify)

SANTERO

Survey on animal health surveillance standards by Santero (http://santero.fp7-risksur.eu/)

Use of existing standards for surveillance

This section enquires about the use and relevance of existing standards for animal health surveillance. With standards, we mean "something considered by an authority or by general consent as an approved model or quality, can serve as a basis of comparison across countries". This can include OIE standards (e.g. OIE surveillance guide), industry guidelines (e.g. private surveillance and monitoring in the pig industry), best practice recommendations (e.g. RISKSUR document), EU regulation (e.g. disease notification rules), national regulation (e.g. enhanced passive surveillance rules for the UK), etc.

* Please read the statements below and tick the appropriate box

	Fully agree	Agree	Neither agree nor disagree	Disagree	Totally disagree	Not applicable
All surveillance activities in my institution are conducted according to best practice	○	○	○	○	○	○
Existing surveillance standards are adequate	○	○	○	○	○	○
Existing guidance on surveillance standards is adequate	○	○	○	○	○	○
In our institution we are aware of surveillance standards, but adoption is limited	○	○	○	○	○	○
Existing surveillance standards are flexible enough for the needs of my institution	○	○	○	○	○	○

Comment field (optional)

* Do public (i.e. government) or private (i.e. industry) standards in your country go beyond regional (e.g. EU) or international (e.g. OIE) standards?

- Yes
- No
- Don't know

Comment field (optional)

* Do you consult the OIE codes for terrestrial and/or aquatic species for your SURVEILLANCE work?

See the screenshots below for guidance or click on these links:

Terrestrial animals: http://www.oie.int/international-standard-setting/terrestrial-code/

Aquatic animals: http://www.oie.int/international-standard-setting/aquatic-code/

◯ Yes

◯ No

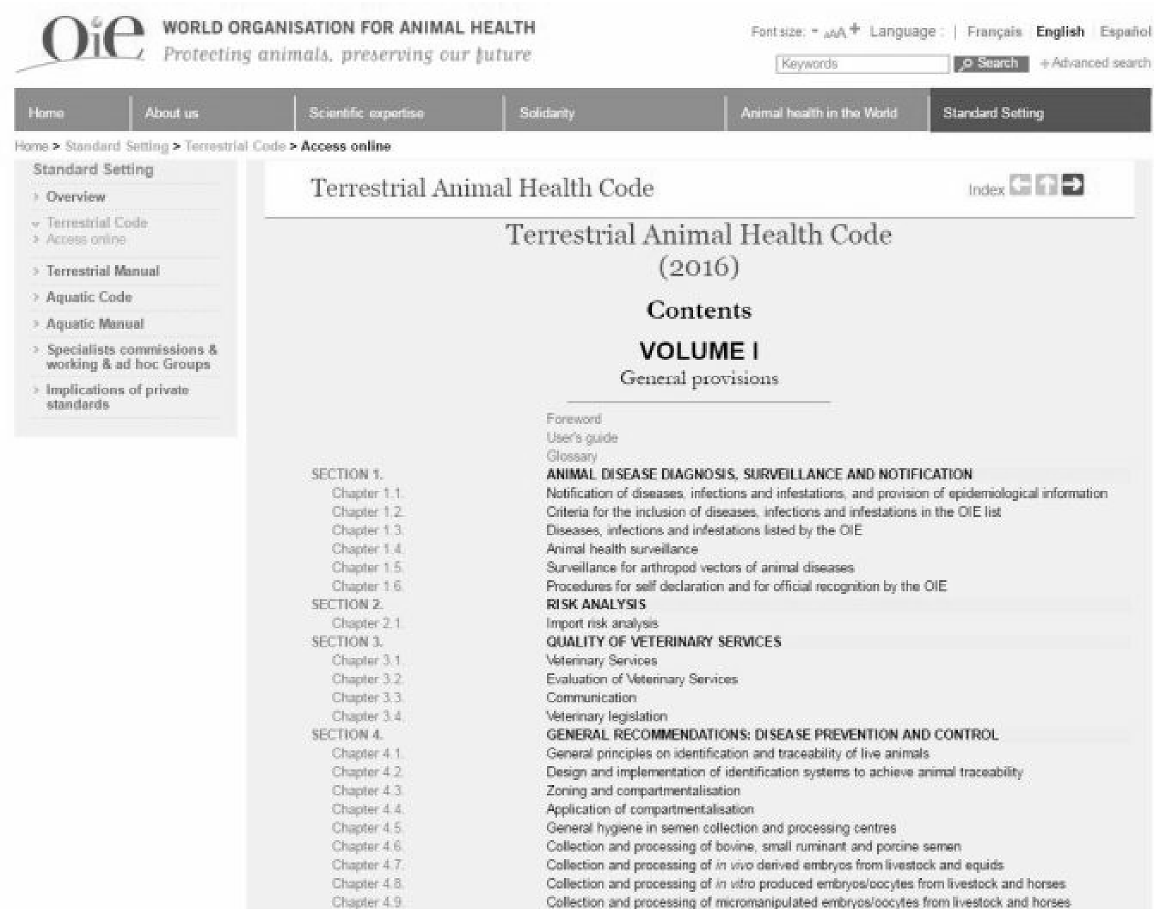

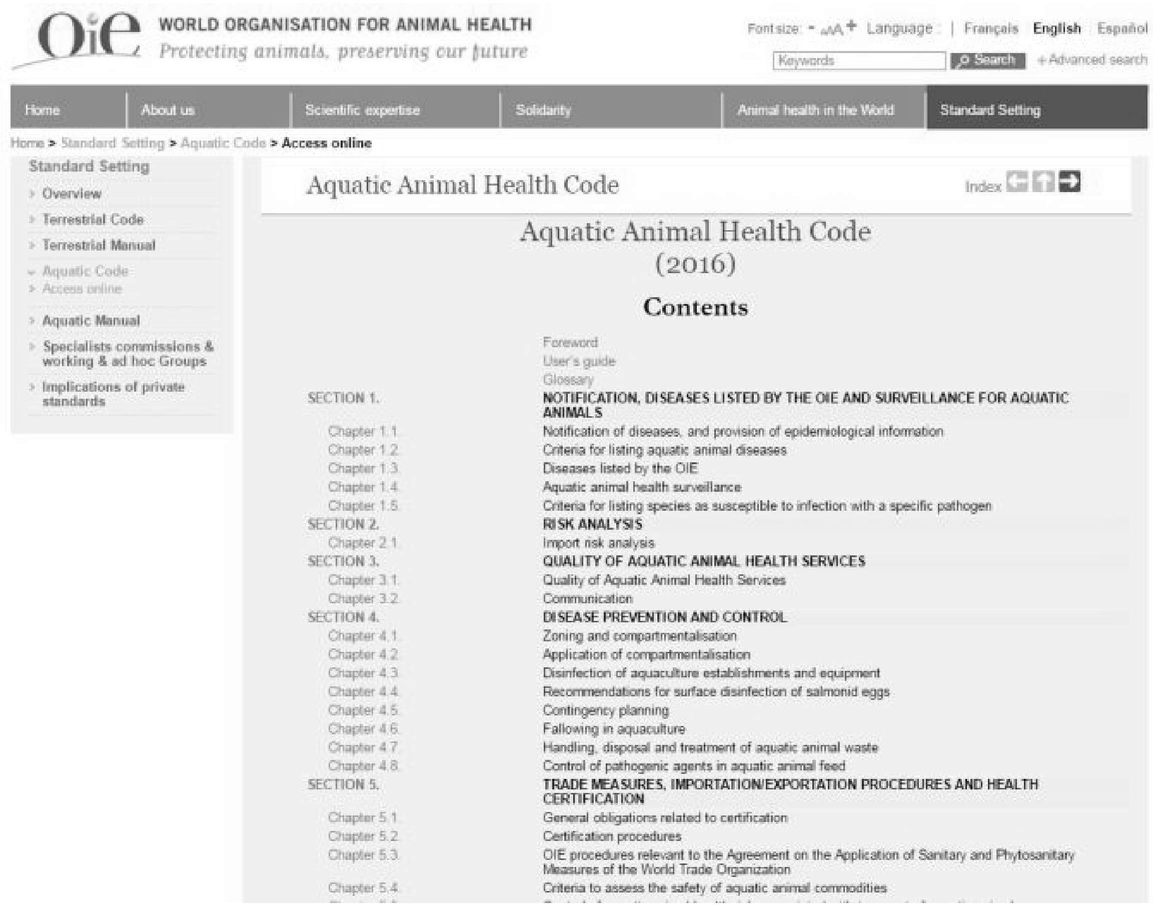

Survey on animal health surveillance standards by Santero (http://santero.fp7-risksur.eu/)

OIE codes for terrestrial and/or aquatic species

* How frequently do you use the OIE codes for terrestrial and/or aquatic species?

- Several times a week
- Several times a month
- Several times a year
- Once a year
- Every few years

Comment field (optional)

* What is the relevance of the OIE codes for terrestrial and/or aquatic species for your surveillance work?

- Very relevant
- Relevant
- Moderately relevant
- Slightly relevant
- Not relevant

Comment field (optional)

* What surveillance related activities do you use the OIE codes for terrestrial or aquatic species for?

Do you use them to get guidance on...

- [ ] Prioritisation of hazards for surveillance
- [ ] Planning of surveillance activities
- [ ] Surveillance design
- [ ] Surveillance implementation
- [ ] Diagnostic procedures for surveillance
- [ ] Surveillance data analysis
- [ ] Surveillance data interpretation
- [ ] Communication/reporting of surveillance findings
- [ ] Evaluation of surveillance
- [ ] Requirements for certification or accreditation
- [ ] Other

* How well do the OIE codes for terrestrial and/or aquatic species fulfil the needs you have in your surveillance work?

- ( ) Very good
- ( ) Good
- ( ) Acceptable
- ( ) Poor
- ( ) Very poor

Comment field (optional)

SANTERO

Survey on animal health surveillance standards by Santero (http://santero.fp7-risksur.eu/)

Use of OIE surveillance guide

* Do you consult the OIE Guide to Terrestrial Animal Health Surveillance for your SURVEILLANCE work?

*See the picture below for guidance or click the link:*http://www.oie.int/for-the-media/press-releases/detail/article/a-new-oie-guide-to-better-surveillance-and-detection-of-health-risks-related-to-animals/

- ◯ Yes
- ◯ No

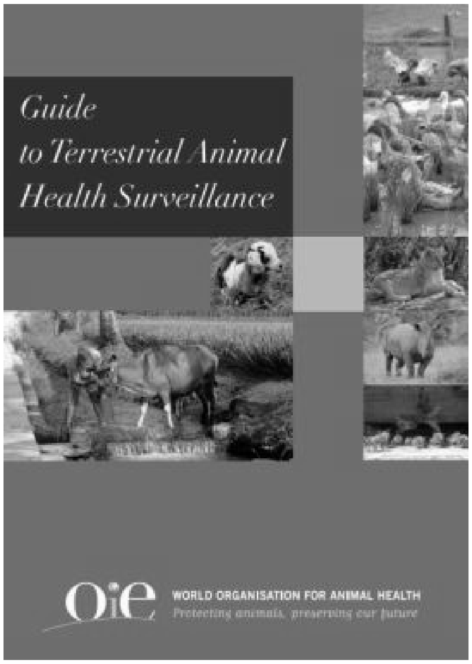

SANTERO

Survey on animal health surveillance standards by Santero (http://santero.fp7-risksur.eu/)

OIE Guide to Terrestrial Animal Health Surveillance

* How frequently do you use the OIE *Guide to Terrestrial Animal Health Surveillance* for your surveillance work?

- Several times a week
- Several times a month
- Several times a year
- Once a year
- Every few years

Comment field (optional)

* What is the relevance of the OIE *Guide to Terrestrial Animal Health Surveillance* for your surveillance work?

- Very relevant
- Relevant
- Moderately relevant
- Slightly relevant
- Not relevant

Comment field (optional)

* What surveillance related activities do you use the OIE *Guide to Terrestrial Animal Health Surveillance* for?

Do you use it to get guidance on...

- ☐ Prioritisation of hazards for surveillance
- ☐ Planning of surveillance activities
- ☐ Surveillance design
- ☐ Surveillance implementation
- ☐ Diagnostic procedures for surveillance
- ☐ Surveillance data analysis
- ☐ Surveillance data interpretation
- ☐ Communication/reporting of surveillance findings
- ☐ Evaluation of surveillance
- ☐ Requirements for certification or accreditation
- ☐ Other

* How well does the OIE *Guide to Terrestrial Animal Health Surveillance* fulfil the needs you have in your surveillance work?

- ○ Very good
- ○ Good
- ○ Acceptable
- ○ Poor
- ○ Very poor

Comment field (optional)

SANTERO

Survey on animal health surveillance standards by Santero (http://santero.fp7-risksur.eu/)

Use of Codex Alimentarius

* Do you consult the Codex Alimentarius for your SURVEILLANCE work?

*See the screenshot below for guidance or click the link:*http://www.fao.org/fao-who-codexalimentarius/en/

- ◯ Yes
- ◯ No

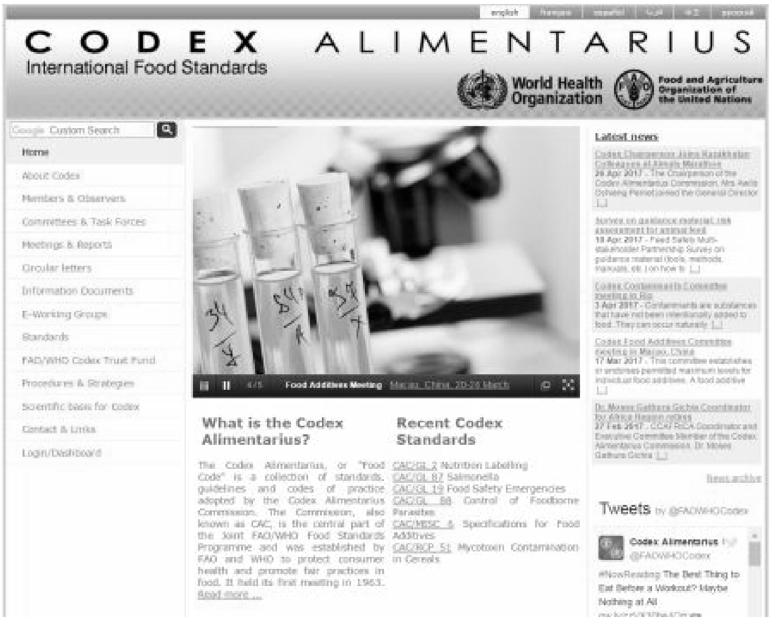

SANTERO

Survey on animal health surveillance standards by Santero (http://santero.fp7-risksur.eu/)

Codex Alimentarius

* How frequently do you use the *Codex Alimentarius* for your surveillance work?

- ◯ Several times a week
- ◯ Several times a month
- ◯ Several times a year
- ◯ Once a year
- ◯ Every few years

Comment field (optional)

* What is the relevance of the *Codex Alimentarius* for your surveillance work?

- ◯ Very relevant
- ◯ Relevant
- ◯ Moderately relevant
- ◯ Slightly relevant
- ◯ Not relevant

Comment field (optional)

* What surveillance related activities do you use the *Codex Alimentarius* for?

Do you use it to get guidance on...

- [ ] Prioritisation of hazards for surveillance
- [ ] Planning of surveillance activities
- [ ] Surveillance design
- [ ] Surveillance implementation
- [ ] Diagnostic procedures for surveillance
- [ ] Surveillance data analysis
- [ ] Surveillance data interpretation
- [ ] Communication/reporting of surveillance findings
- [ ] Evaluation of surveillance
- [ ] Requirements for certification or accreditation
- [ ] Other

* How well does the *Codex Alimentarius* fulfil the needs you have in your surveillance work?

- ( ) Very good
- ( ) Good
- ( ) Acceptable
- ( ) Poor
- ( ) Very poor

Comment field (optional)

Use of EU legislation

* Do you consult EU legislation for your SURVEILLANCE work?

*See the screenshot below for guidance or click the link:*http://eur-lex.europa.eu/homepage.html

- ◯ Yes
- ◯ No

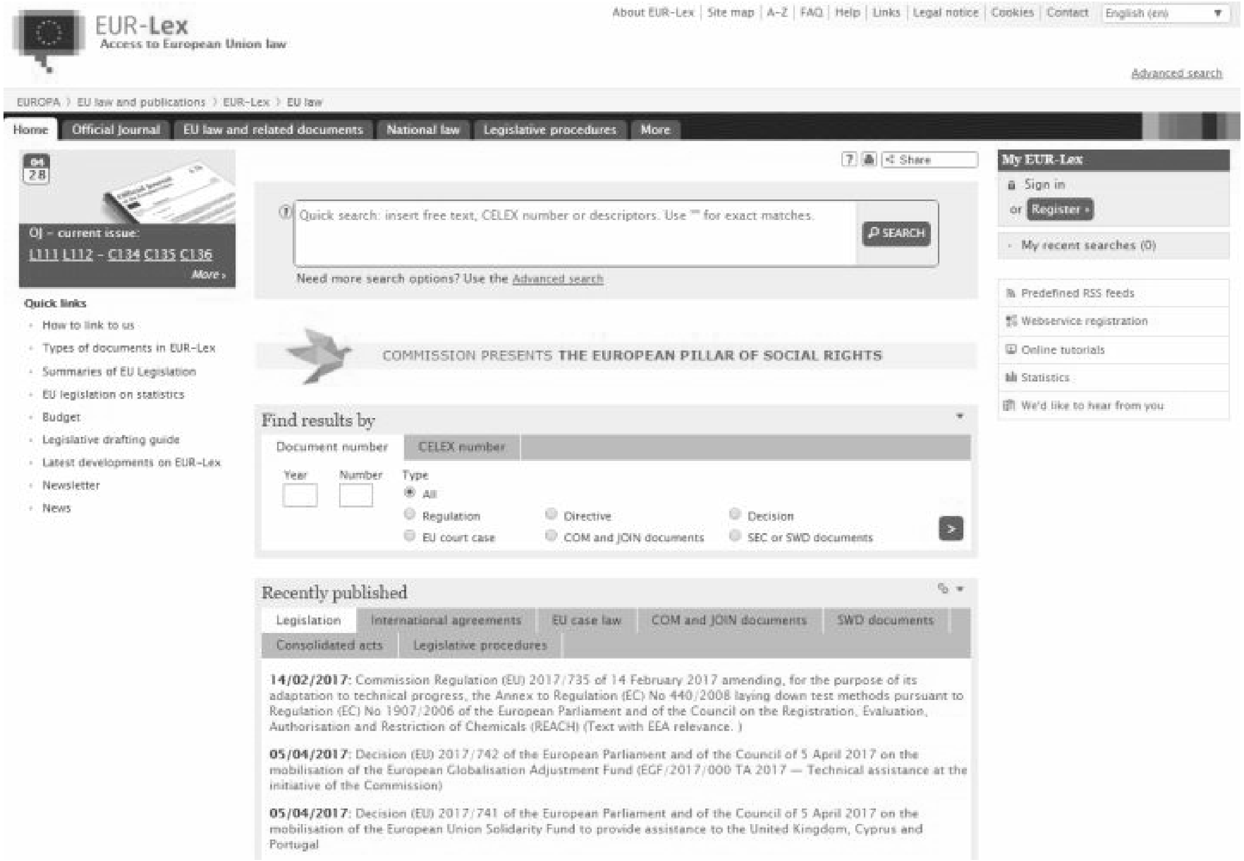

SANTERO

Survey on animal health surveillance standards by Santero (http://santero.fp7-risksur.eu/)

EU legislation

* How frequently do you use EU legislation for your surveillance work?

- ◯ Several times a week
- ◯ Several times a month
- ◯ Several times a year
- ◯ Once a year
- ◯ Every few years

Comment field (optional)

* What is the relevance of EU legislation for your surveillance work?

- ◯ Very relevant
- ◯ Relevant
- ◯ Moderately relevant
- ◯ Slightly relevant
- ◯ Not relevant

Comment field (optional)

* What surveillance related activities do you use EU legislation for?

Do you use them to get guidance on...

- [ ] Prioritisation of hazards for surveillance
- [ ] Planning of surveillance activities
- [ ] Surveillance design
- [ ] Surveillance implementation
- [ ] Diagnostic procedures for surveillance
- [ ] Surveillance data analysis
- [ ] Surveillance data interpretation
- [ ] Communication/reporting of surveillance findings
- [ ] Evaluation of surveillance
- [ ] Requirements for certification or accreditation
- [ ] Other

* How well does the EU legislation fulfil the needs you have in your surveillance work?

- ○ Very good
- ○ Good
- ○ Acceptable
- ○ Poor
- ○ Very poor

Comment field (optional)

Use of national legislation

* Do you consult national legislation (beyond EU law) for your SURVEILLANCE work?

Here we refer to national legislation, which differs from EU legislation, i.e. legislation beyond or different from implementation of EU law or bilateral agreements

- ○ Yes
- ○ No

SANTERO

Survey on animal health surveillance standards by Santero (http://santero.fp7-risksur.eu/)

National legislation

* How frequently do you use national legislation (that differs from EU law)?

- ○ Several times a week
- ○ Several times a month
- ○ Several times a year
- ○ Once a year
- ○ Every few years

Comment field (optional)

* What is the relevance of national legislation (that differs from EU law) for your usual surveillance work?

- ◯ Very relevant
- ◯ Relevant
- ◯ Moderately relevant
- ◯ Slightly relevant
- ◯ Not relevant

Comment field (optional)

* What surveillance related activities do you use the national legislation (that differs from EU law) for?

Do you use it to get guidance on...

- ☐ Prioritisation of hazards for surveillance
- ☐ Planning of surveillance activities
- ☐ Surveillance design
- ☐ Surveillance implementation
- ☐ Diagnostic procedures for surveillance
- ☐ Surveillance data analysis
- ☐ Surveillance data interpretation
- ☐ Communication/reporting of surveillance findings
- ☐ Evaluation of surveillance
- ☐ Requirements for certification or accreditation
- ☐ Other

* How well does the national legislation (that differs from EU law) fulfil the needs you have in your surveillance work?

- Very good
- Good
- Acceptable
- Poor
- Very poor

Comment field (optional)

SANTERO

Survey on animal health surveillance standards by Santero (http://santero.fp7-risksur.eu/)

Use of private industry standards

* Do you consult private industry standards for your SURVEILLANCE work?

- Yes
- No

SANTERO

Survey on animal health surveillance standards by Santero (http://santero.fp7-risksur.eu/)

Private industry standards for surveillance

* How frequently do you use private industry standards for your surveillance work?

- ◯ Several times a week
- ◯ Several times a month
- ◯ Several times a year
- ◯ Once a year
- ◯ Every few years

Comment field (optional)

* What is the relevance of private industry standards for your surveillance work?

- ◯ Very relevant
- ◯ Relevant
- ◯ Moderately relevant
- ◯ Slightly relevant
- ◯ Not relevant

Comment field (optional)

* What surveillance related activities do you use private industry standards for?

Do you use them to get guidance on...

- [ ] Prioritisation of hazards for surveillance
- [ ] Planning of surveillance activities
- [ ] Surveillance design
- [ ] Surveillance implementation
- [ ] Diagnostic procedures for surveillance
- [ ] Surveillance data analysis
- [ ] Surveillance data interpretation
- [ ] Communication/reporting of surveillance findings
- [ ] Evaluation of surveillance
- [ ] Requirements for certification or accreditation
- [ ] Other

* How well do private industry standards fulfil the needs you have in your surveillance work?

- ( ) Very good
- ( ) Good
- ( ) Acceptable
- ( ) Poor
- ( ) Very poor

Comment field (optional)

SANTERO

Use of RISKSUR best practice document

* Do you consult the RISKSUR best practice document for your SURVEILLANCE work?

*See the picture below for guidance or click on the link:*http://www.fp7-risksur.eu/progress/best-practice-document

- ◯ Yes
- ◯ No

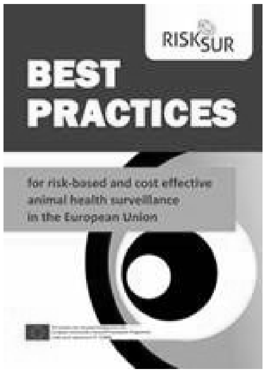

Survey on animal health surveillance standards by Santero (http://santero.fp7-risksur.eu/)

RISKSUR best practice document

* How frequently do you use the RISKSUR best practice document?

- ◯ Several times a week
- ◯ Several times a month
- ◯ Several times a year
- ◯ Once a year
- ◯ Every few years

Comment field (optional)

* What is the relevance of the RISKSUR best practice document for your surveillance work?

- ◯ Very relevant
- ◯ Relevant
- ◯ Moderately relevant
- ◯ Slightly relevant
- ◯ Not relevant

Comment field (optional)

* What surveillance related activities do you use the RISKSUR best practice document for?

Do you use it to get guidance on...

- [ ] Prioritisation of hazards for surveillance
- [ ] Planning of surveillance activities
- [ ] Surveillance design
- [ ] Surveillance implementation
- [ ] Diagnostic procedures for surveillance
- [ ] Surveillance data analysis
- [ ] Surveillance data interpretation
- [ ] Communication/reporting of surveillance findings
- [ ] Evaluation of surveillance
- [ ] Requirements for certification or accreditation
- [ ] Other

* How well does the RISKSUR best practice document fulfil the needs you have in your surveillance work?

- ○ Very good
- ○ Good
- ○ Acceptable
- ○ Poor
- ○ Very poor

Comment field (optional)

SANTERO

Use of FAO risk-based disease surveillance manual

* Do you consult the FAO 2014 manual "Risk-based disease surveillance – A manual for veterinarians on the design and analysis of surveillance for demonstration of freedom from disease" for your SURVEILLANCE work?

See the picture below for guidance or click on the link: http://www.fao.org/3/a-i4205e.pdf

- ◯ Yes
- ◯ No

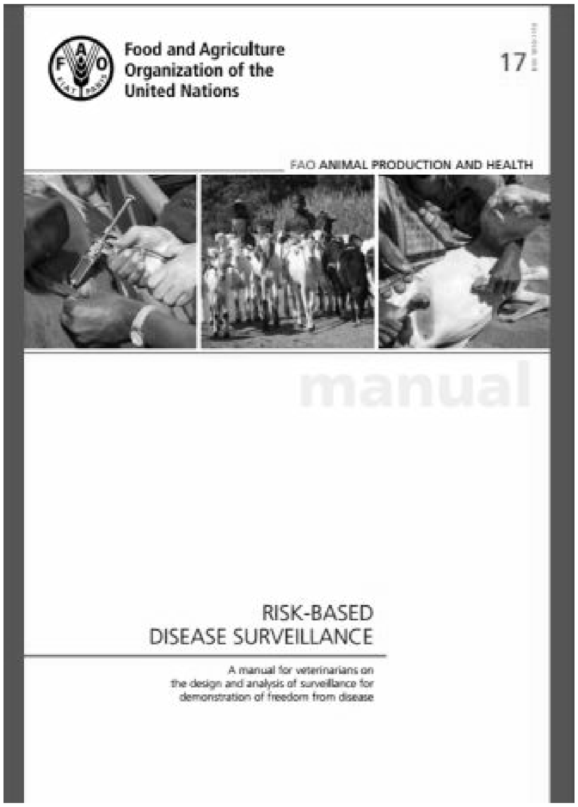

* How frequently do you use the FAO 2014 manual "risk-based disease surveillance..."?

- ◯ Several times a week
- ◯ Several times a month
- ◯ Several times a year
- ◯ Once a year
- ◯ Every few years

Comment field (optional)

* What is the relevance of the FAO 2014 manual "risk-based disease surveillance..." for your surveillance work?

- ◯ Very relevant
- ◯ Relevant
- ◯ Moderately relevant
- ◯ Slightly relevant
- ◯ Not relevant

Comment field (optional)

* What surveillance related activities do you use the FAO 2014 manual "risk-based disease surveillance..." for?

Do you use it to get guidance on...

- [ ] Prioritisation of hazards for surveillance
- [ ] Planning of surveillance activities
- [ ] Surveillance design
- [ ] Surveillance implementation
- [ ] Diagnostic procedures for surveillance
- [ ] Surveillance data analysis
- [ ] Surveillance data interpretation
- [ ] Communication/reporting of surveillance findings
- [ ] Evaluation of surveillance
- [ ] Requirements for certification or accreditation
- [ ] Other

* How well does the FAO 2014 manual "risk-based disease surveillance..." fulfil the needs you have in your surveillance work?

- ( ) Very good
- ( ) Good
- ( ) Acceptable
- ( ) Poor
- ( ) Very poor

Comment field (optional)

SANTERO

Survey on animal health surveillance standards by Santero (http://santero.fp7-risksur.eu/)

Use of book "Epidemiological surveillance in animal health"

* Do you consult the book "Epidemiological surveillance in animal health" by CIRAD, AEEMA, FAO and OIE for your SURVEILLANCE work?

*See the picture below for guidance or click on the link:*http://oie.int/doc/en_document.php?numrec=3741703

- ◯ Yes
- ◯ No

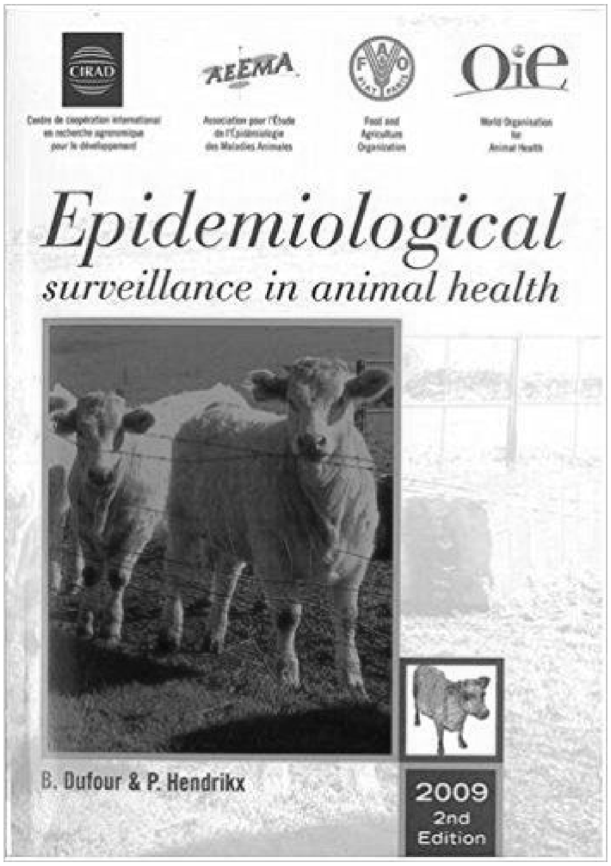

Survey on animal health surveillance standards by Santero (http://santero.fp7-risksur.eu/)

Book "Epidemiological surveillance in animal health"

* How frequently do you use the book *Epidemiological surveillance in animal health* for your surveillance work?

- ◯ Several times a week
- ◯ Several times a month
- ◯ Several times a year
- ◯ Once a year
- ◯ Every few years

Comment field (optional)

* What is the relevance of the book *Epidemiological surveillance in animal health* for your surveillance work?

- ◯ Very relevant
- ◯ Relevant
- ◯ Moderately relevant
- ◯ Slightly relevant
- ◯ Not relevant

Comment field (optional)

* What surveillance related activities do you use the book *Epidemiological surveillance in animal health* for?

Do you use it to get guidance on...

- ☐ Prioritisation of hazards for surveillance
- ☐ Planning of surveillance activities
- ☐ Surveillance design
- ☐ Surveillance implementation
- ☐ Diagnostic procedures for surveillance
- ☐ Surveillance data analysis
- ☐ Surveillance data interpretation
- ☐ Communication/reporting of surveillance findings
- ☐ Evaluation of surveillance
- ☐ Requirements for certification or accreditation
- ☐ Other

* How well does the book *Epidemiological surveillance in animal health* fulfil the needs you have in your surveillance work?

- ○ Very good
- ○ Good
- ○ Acceptable
- ○ Poor
- ○ Very poor

Comment field (optional)

SANTERO

Survey on animal health surveillance standards by Santero (http://santero.fp7-risksur.eu/)

Use of peer-reviewed publications

* Do you consult peer-reviewed scientific publications for your SURVEILLANCE work?

- ◯ Yes
- ◯ No

SANTERO

Survey on animal health surveillance standards by Santero (http://santero.fp7-risksur.eu/)

Peer-reviewed publications on surveillance

* How frequently do you use peer-reviewed scientific publications on surveillance for your surveillance work?

- ◯ Several times a week
- ◯ Several times a month
- ◯ Several times a year
- ◯ Once a year
- ◯ Every few years

Comment field (optional)

* What is the relevance of the peer-reviewed scientific publications on surveillance for your surveillance work?

- ◯ Very relevant
- ◯ Relevant
- ◯ Moderately relevant
- ◯ Slightly relevant
- ◯ Not relevant

Comment field (optional)

* What surveillance related activities do you use peer-reviewed scientific publications on surveillance for?

Do you use them to get guidance on...

- ☐ Prioritisation of hazards for surveillance
- ☐ Planning of surveillance activities
- ☐ Surveillance design
- ☐ Surveillance implementation
- ☐ Diagnostic procedures for surveillance
- ☐ Surveillance data analysis
- ☐ Surveillance data interpretation
- ☐ Communication/reporting of surveillance findings
- ☐ Evaluation of surveillance
- ☐ Requirements for certification or accreditation

Other

* How well do peer-reviewed scientific publications on surveillance fulfil the needs you have in your surveillance work?

- Very good
- Good
- Acceptable
- Poor
- Very poor

Comment field (optional)

SANTERO

Survey on animal health surveillance standards by Santero (http://santero.fp7-risksur.eu/)

Use of FAO technical paper surveillance and zoning for aquatic animal diseases

* Do you consult the FAO technical paper "surveillance and zoning for aquatic animal diseases" for your SURVEILLANCE work?

*See the picture below for guidance or click on link:*http://www.fao.org/docrep/007/y5325e/y5325e00.htm

- Yes
- No

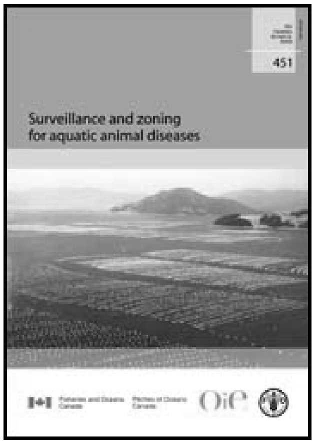

SANTERO

Survey on animal health surveillance standards by Santero (http://santero.fp7-risksur.eu/)

FAO technical paper surveillance and zoning for aquatic animal diseases

* How frequently do you use the FAO technical paper *Surveillance and Zoning for Aquatic Animal Diseases* for your surveillance work?

- ○ Several times a week
- ○ Several times a month
- ○ Several times a year
- ○ Once a year
- ○ Every few years

Comment field (optional)

* What is the relevance of the FAO technical paper*Surveillance and Zoning for Aquatic Animal Diseases* for your surveillance work?

- ○ Very relevant
- ○ Relevant
- ○ Moderately relevant
- ○ Slightly relevant
- ○ Not relevant

Comment field (optional)

[ ]

* What surveillance related activities do you use FAO technical paper*Surveillance and Zoning for Aquatic Animal Diseases* for?

Do you use them to get guidance on...

- [ ] Prioritisation of hazards for surveillance
- [ ] Planning of surveillance activities
- [ ] Surveillance design
- [ ] Surveillance implementation
- [ ] Diagnostic procedures for surveillance
- [ ] Surveillance data analysis
- [ ] Surveillance data interpretation
- [ ] Communication/reporting of surveillance findings
- [ ] Evaluation of surveillance
- [ ] Requirements for certification or accreditation

Other

[ ]

* How well does the FAO technical paper *Surveillance and Zoning for Aquatic Animal Diseases* fulfil the needs you have in your surveillance work?

- ◯ Very good
- ◯ Good
- ◯ Acceptable
- ◯ Poor
- ◯ Very poor

Comment field (optional)

SANTERO

Survey on animal health surveillance standards by Santero (http://santero.fp7-risksur.eu/)

Use of OIE Guide for Aquatic Animal Health surveillance

* Do you consult the OIE Guide for Aquatic Animal Health surveillance for your SURVEILLANCE work?
*See the screenshot below for guidance or click on link:*http://www.oie.int/doc/ged/d6714.pdf

- ◯ Yes
- ◯ No

Guide for Aquatic Animal Health Surveillance

Primary authors:

Flavio Corsin
Aquaculture and Aquatic Animal Health Specialist
Hanoi
Vietnam

Marios Georgiadis
Lecturer of Epidemiology
Faculty of Veterinary Medicine
Aristotle University of Thessaloniki
Greece

K. Larry Hammell
Professor, Dept of Health Management
Director, AVC Centre for Aquatic Health Sciences Atlantic Veterinary College
University of Prince Edward Island
Canada

SANTERO

**Survey on animal health surveillance standards by Santero** (http://santero.fp7-risksur.eu/)
OIE Guide For Aquatic Animal Health Surveillance

* How frequently do you use the *OIE Guide For Aquatic Animal Health Surveillance* for your surveillance work?

- ◯ Several times a week
- ◯ Several times a month
- ◯ Several times a year
- ◯ Once a year
- ◯ Every few years

Comment field (optional)

* What is the relevance of the *OIE Guide For Aquatic Animal Health Surveillance* for your surveillance work?

- ◯ Very relevant
- ◯ Relevant
- ◯ Moderately relevant
- ◯ Slightly relevant
- ◯ Not relevant

Comment field (optional)

* What surveillance related activities do you use the *OIE Guide For Aquatic Animal Health Surveillance* for?

Do you use them to get guidance on...

- [ ] Prioritisation of hazards for surveillance
- [ ] Planning of surveillance activities
- [ ] Surveillance design
- [ ] Surveillance implementation
- [ ] Diagnostic procedures for surveillance
- [ ] Surveillance data analysis
- [ ] Surveillance data interpretation
- [ ] Communication/reporting of surveillance findings
- [ ] Evaluation of surveillance
- [ ] Requirements for certification or accreditation

Other

* How well does the *OIE Guide For Aquatic Animal Health Surveillance* fulfil the needs you have in your surveillance work?

- ○ Very good
- ○ Good
- ○ Acceptable
- ○ Poor
- ○ Very poor

Comment field (optional)

SANTERO

Survey on animal health surveillance standards by Santero (http://santero.fp7-risksur.eu/)

Use of book *Survey toolbox for aquatic animal diseases: a practical manual and software package*

* Do you consult the book *Survey toolbox for aquatic animal diseases: a practical manual and software package* for your SURVEILLANCE work?

See the picture below for guidance or click on link: http://aciar.gov.au/publication/mn094

- ◯ Yes
- ◯ No

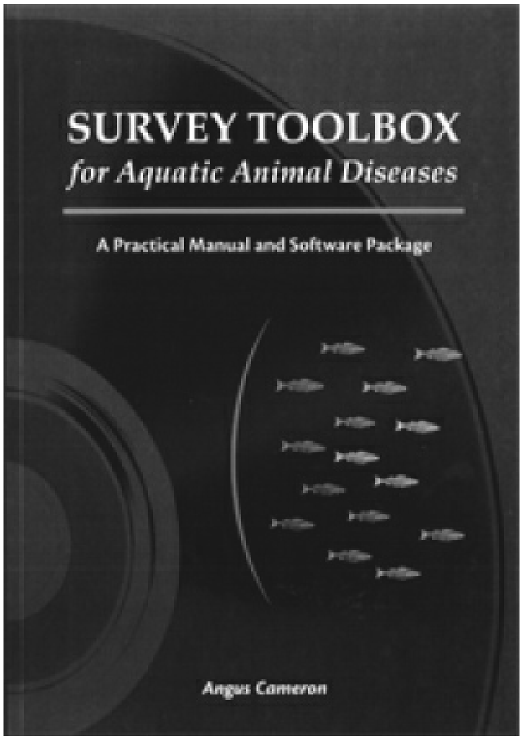

SANTERO

Survey on animal health surveillance standards by Santero (http://santero.fp7-risksur.eu/)

Survey toolbox for aquatic animal diseases: a practical manual

* How frequently do you use the *Survey toolbox for aquatic animal diseases: a practical manual* for your surveillance work?

- Several times a week
- Several times a month
- Several times a year
- Once a year
- Every few years

Comment field (optional)

* What is the relevance of the *Survey toolbox for aquatic animal diseases: a practical manual* for your surveillance work?

- Very relevant
- Relevant
- Moderately relevant
- Slightly relevant
- Not relevant

Comment field (optional)

* What surveillance related activities do you use the *Survey toolbox for aquatic animal diseases: a practical manual* for?

Do you use them to get guidance on...

- ☐ Prioritisation of hazards for surveillance
- ☐ Planning of surveillance activities
- ☐ Surveillance design
- ☐ Surveillance implementation
- ☐ Diagnostic procedures for surveillance
- ☐ Surveillance data analysis
- ☐ Surveillance data interpretation
- ☐ Communication/reporting of surveillance findings
- ☐ Evaluation of surveillance
- ☐ Requirements for certification or accreditation

Other

* How well does the *Survey toolbox for aquatic animal diseases: a practical manual* fulfil the needs you have in your surveillance work?

- ◯ Very good
- ◯ Good
- ◯ Acceptable
- ◯ Poor
- ◯ Very poor

Comment field (optional)

SANTERO

Survey on animal health surveillance standards by Santero (http://santero.fp7-risksur.eu/)

Use of further surveillance standards

* Do you consult further surveillance standards (not listed so far) for your SURVEILLANCE work?

- ◯ Yes
- ◯ No

SANTERO

Survey on animal health surveillance standards by Santero (http://santero.fp7-risksur.eu/)

Other surveillance standards

* Please describe what other surveillance standards you use, what the frequency of use is, their relevance, whether they fulfil your needs and what purposes you use them for

SANTERO

Survey on animal health surveillance standards by Santero (http://santero.fp7-risksur.eu/)

Information and data exchange

This section enquires about procedures for data and information sharing and learning, both formal and informal

* How often do you use the following sources to learn about new surveillance standards or best practice for surveillance?

International sources

	Very often	Often	Sometimes	Rarely	Never	Not applicable
International conference or symposium	◯	◯	◯	◯	◯	◯
International scientific publications	◯	◯	◯	◯	◯	◯
International lay publications	◯	◯	◯	◯	◯	◯
Official communications by OIE or FAO	◯	◯	◯	◯	◯	◯
Official communications by private standard setting bodies	◯	◯	◯	◯	◯	◯
EU bulletin	◯	◯	◯	◯	◯	◯
International training event	◯	◯	◯	◯	◯	◯
Exchange with international colleagues outside my workplace	◯	◯	◯	◯	◯	◯
Collaboration in international surveillance research or projects	◯	◯	◯	◯	◯	◯
International online courses	◯	◯	◯	◯	◯	◯

Comments/explanations/other

*

National sources

	Very often	Often	Sometimes	Rarely	Never	Not applicable
National conference or symposium	◯	◯	◯	◯	◯	◯
Scientific national publications	◯	◯	◯	◯	◯	◯
Lay national publications	◯	◯	◯	◯	◯	◯
Official communications by private standard setting bodies	◯	◯	◯	◯	◯	◯
Official communications by national public bodies	◯	◯	◯	◯	◯	◯
National, non-institutional training event	◯	◯	◯	◯	◯	◯
Institutional training event	◯	◯	◯	◯	◯	◯
Exchange with colleagues at my workplace	◯	◯	◯	◯	◯	◯
Exchange with national colleagues outside my workplace	◯	◯	◯	◯	◯	◯
Collaboration in national surveillance research or projects	◯	◯	◯	◯	◯	◯
National online courses	◯	◯	◯	◯	◯	◯

Comments/explanations/other

* Do you feel sufficiently informed about existing surveillance standards and best practice?

◯ Yes

◯ No

◯ Don't know

If no, what is missing? Please describe

* Do you receive information on new surveillance standards and best practice in a timely manner?

- Yes
- No
- Don't know

If no, what is missing? Please describe

Please add any suggestions you may have on how information sharing or access to information on surveillance standards or best practice could be improved?

SANTERO

Survey on animal health surveillance standards by Santero (http://santero.fp7-risksur.eu/)

Adoption of new surveillance standards and evaluation

This section enquires about drivers and hindering factors for the adoption of new surveillance standards and evaluation

* How relevant are considerations of cost-effectiveness when making a decision to adopt new surveillance standards?

Absolutely essential	Very important	Of average importance	Of little importance	Not important at all
○	○	○	○	○

* When new surveillance standards become available, how is the economic efficiency of the potential change in surveillance commonly evaluated in your institution?

- [ ] No formal evaluation
- [ ] Quantitative assessment of costs of the change in surveillance
- [ ] Quantitative assessment of the effectiveness of the change in surveillance (e.g. timeliness, sensitivity, acceptability)
- [ ] Quantitative cost-benefit analysis
- [ ] Descriptive assessment of consequences
- [ ] Descriptive assessment of costs
- [ ] Quantitative cost-effectiveness analysis
- [ ] Other (please specify)

* Please rate the availability of the following resources for the adoption of new surveillance standards in your institution?

	Insufficient	Somewhat insufficient	Neither insufficient nor sufficient	Somewhat sufficient	Sufficient	Don't know
Technical epidemiological skills	◯	◯	◯	◯	◯	◯
Economics skills	◯	◯	◯	◯	◯	◯
Evaluation knwoledge	◯	◯	◯	◯	◯	◯
Human resource (i.e. labour)	◯	◯	◯	◯	◯	◯
Formal guidance on approaches and methods	◯	◯	◯	◯	◯	◯
Processes for structured information exchange	◯	◯	◯	◯	◯	◯
Financial resources	◯	◯	◯	◯	◯	◯
Processes for learning	◯	◯	◯	◯	◯	◯
Time	◯	◯	◯	◯	◯	◯

Comments/explanations/other

* Please rate the availability of the following resources for the adoption of evaluation standards for surveillance in your institution?

	Insufficient	Somewhat insufficient	Neither insufficient nor sufficient	Somewhat sufficient	Sufficient	Don't know
Technical epidemiological skills	◯	◯	◯	◯	◯	◯
Economics skills	◯	◯	◯	◯	◯	◯
Evaluation knowledge	◯	◯	◯	◯	◯	◯
Human resource (i.e. labour)	◯	◯	◯	◯	◯	◯
Formal guidance on approaches and methods	◯	◯	◯	◯	◯	◯
Processes for structured information exchange	◯	◯	◯	◯	◯	◯
Financial resources	◯	◯	◯	◯	◯	◯
Processes for learning	◯	◯	◯	◯	◯	◯
Time	◯	◯	◯	◯	◯	◯

Comments/explanations/other

* In your opinion, is there a need for international evaluation standards for surveillance?

◯ Yes

◯ No

◯ Don't know

* Please explain your answer

* Who should be in charge of developing such evaluation standards?

- [ ] EU
- [ ] OIE
- [ ] FAO
- [ ] WTO
- [ ] Codex Alimentarius Commission
- [ ] Scientific community

Other and/or explanations

* What should these surveillance evaluation standards entail?

* Do you perform assessments or evaluations of surveillance?

- ( ) Yes
- ( ) No

SANTERO

Survey on animal health surveillance standards by Santero (http://santero.fp7-risksur.eu/)

Capability to assess surveillance attributes / characteristics

* How confident do you feel in your capability to assess the following surveillance attributes?

Please refer to the RISKSUR glossary for definitions of these attributes: http://www.fp7-risksur.eu/terminology/glossary

	Very confident	Confident	Neutral	Not very confident	Not at all confident	Not applicable
Sensitivity (effectiveness attribute)	○	○	○	○	○	○
Timeliness (effectiveness attribute)	○	○	○	○	○	○
False alarm rate (effectiveness attribute)	○	○	○	○	○	○
Representativeness (effectiveness attribute)	○	○	○	○	○	○
Coverage (effectiveness attribute)	○	○	○	○	○	○
Precision (effectiveness attribute)	○	○	○	○	○	○
Bias (effectiveness attribute)	○	○	○	○	○	○
Robustness (effectiveness attribute)	○	○	○	○	○	○
Negative predictive value (effectiveness attribute)	○	○	○	○	○	○
Positive predictive value (effectiveness attribute)	○	○	○	○	○	○
Acceptability (functional attribute)	○	○	○	○	○	○
Availability (functional attribute)	○	○	○	○	○	○
Sustainability (functional attribute)	○	○	○	○	○	○
Compatibility (functional attribute)	○	○	○	○	○	○
Multiple hazard (functional attribute)	○	○	○	○	○	○
Flexibility (functional attribute)	○	○	○	○	○	○
Simplicity (functional attribute)	○	○	○	○	○	○
Surveillance system organisation (functional attribute)	○	○	○	○	○	○
Cost-benefit criterion (e.g. BCR, NPV, IRR)	○	○	○	○	○	○
Cost-effectiveness criterion (e.g. ICER, ACER)	○	○	○	○	○	○

	Very confident	Confident	Neutral	Not very confident	Not at all confident	Not applicable
Cost	○	○	○	○	○	○
Benefit	○	○	○	○	○	○
Cost of error	○	○	○	○	○	○

Comments/explanations/other

SANTERO

Survey on animal health surveillance standards by Santero (http://santero.fp7-risksur.eu/)

Future developments and needs

A scoping survey revealed that there is a need for more standardised, but flexible surveillance approaches to enable the comparison across different countries. Furthermore, recent findings of the RISKSUR project (http://www.fp7-risksur.eu/) demonstrated a lack of transparency in the description of how surveillance outputs and information are obtained. This section aims to assess how surveillance outputs/information are currently communicated to inform the harmonisation of reporting guidelines.

* Does your institution produce (or is your institution responsible for producing) reports on the results/findings of surveillance or information about surveillance activities?

In this section, when we use the term "reporting" of surveillance outcomes, we refer to communication of surveillance to the public, not to reporting of compulsory/official information

○ Yes

○ No

SANTERO

Survey on animal health surveillance standards by Santero (http://santero.fp7-risksur.eu/)

Communication of surveillance activities and findings

* In which form do you report/communicate surveillance activities or findings?

- [ ] Internal reports
- [ ] Publicly available reports
- [ ] Other (please specify)

* What type of reference/standard/guidelines do you use to guide this reporting/communication?

- [ ] In my institution, we do not use such guidelines
- [ ] Guidelines specific to my institution
- [ ] Pre-defined document/guidelines
- [ ] STROBE-Vet or similar guidelines
- [ ] Other (please specify and - if appropriate - provide a link)

* What type of information do you commonly report?

- [ ] Background/contextual information
- [ ] Description of surveillance component
- [ ] Target population
- [ ] Suspicion of disease
- [ ] Enhancements
- [ ] Testing protocols
- [ ] Study design
- [ ] Sampling strategy
- [ ] How the data are generated
- [ ] How the data transferred
- [ ] How the data are manipulated
- [ ] Data analysis
- [ ] Results/findings
- [ ] Interpretation of findings
- [ ] References
- [ ] Other (please specify)

SANTERO

Survey on animal health surveillance standards by Santero (http://santero.fp7-risksur.eu/)

Surveillance reporting by others

* How important are these items when you look at surveillance activities or findings reported/produced by others?

	Very relevant	Relevant	Moderately relevant	Slightly relevant	Not relevant	Not applicable
Background/contextual information	○	○	○	○	○	○
Description of surveillance component	○	○	○	○	○	○
Target population	○	○	○	○	○	○
Suspicion of disease	○	○	○	○	○	○
Enhancements	○	○	○	○	○	○
Testing protocols	○	○	○	○	○	○
Study design	○	○	○	○	○	○
Sampling strategy	○	○	○	○	○	○
How the data are generated	○	○	○	○	○	○
How the data transferred	○	○	○	○	○	○
How the data are manipulated	○	○	○	○	○	○
Data analysis	○	○	○	○	○	○
Results/findings	○	○	○	○	○	○
Interpretation of findings	○	○	○	○	○	○
References	○	○	○	○	○	○

Comments/explanations/other

* How difficult would it be for you if reporting/communicating on these elements became compulsory?

	Very difficult	Difficult	Moderately difficult	Slightly difficult	Not difficult	Not applicable
Background/contextual information	◯	◯	◯	◯	◯	◯
Description of surveillance component	◯	◯	◯	◯	◯	◯
Target population	◯	◯	◯	◯	◯	◯
Suspicion of disease	◯	◯	◯	◯	◯	◯
Enhancements	◯	◯	◯	◯	◯	◯
Testing protocols	◯	◯	◯	◯	◯	◯
Study design	◯	◯	◯	◯	◯	◯
Sampling strategy	◯	◯	◯	◯	◯	◯
How the data are generated	◯	◯	◯	◯	◯	◯
How the data transferred	◯	◯	◯	◯	◯	◯
How the data are manipulated	◯	◯	◯	◯	◯	◯
Data analysis	◯	◯	◯	◯	◯	◯
Results/findings	◯	◯	◯	◯	◯	◯
Interpretation of findings	◯	◯	◯	◯	◯	◯
References	◯	◯	◯	◯	◯	◯

Comments/explanations/other

* If there were guidelines on reporting outcomes of surveillance, what is the likelihood that you would use them?

- ◯ Definitely
- ◯ Probably yes
- ◯ Possibly
- ◯ Probably not
- ◯ Definitely not

Comment field (optional)

SANTERO

Survey on animal health surveillance standards by Santero (http://santero.fp7-risksur.eu/)

Contact information

* In case we would like to use one of your quotes, which would be done in an anonymous way by removing all information that could identify you, would you like to review the quote before publication?

- ◯ You can use my quotes, but I would like to review them
- ◯ You can use my quotes, I don't need to review them
- ◯ Please do not use any of my quotes

If you ticked that you would like to review your quote, please provide your email address so that we can send you any of your quotes we may want to use for review

Survey on animal health surveillance standards by Santero (http://santero.fp7-risksur.eu/)

End of survey

Thank you very much for your participation in this survey! Should you have any questions, please send an email to Maria Garza at mgarza3@rvc.ac.uk or Barbara Haesler at bhaesler@rvc.ac.uk
